# An experimentally induced osteoarthritis model in horses performed on both metacarpophalangeal and metatarsophalangeal joints: Technical, clinical, imaging, biochemical, macroscopic and microscopic characterization

**DOI:** 10.1371/journal.pone.0235251

**Published:** 2020-06-25

**Authors:** Lélia Bertoni, Sandrine Jacquet-Guibon, Thomas Branly, Florence Legendre, Mélanie Desancé, Céline Mespoulhes, Martine Melin, Daniel-Jean Hartmann, Amandine Schmutz, Jean-Marie Denoix, Philippe Galéra, Magali Demoor, Fabrice Audigié

**Affiliations:** 1 CIRALE, USC 957, BPLC, INRA, Ecole Nationale Vétérinaire d'Alfort, Maisons-Alfort, France; 2 NORMANDIE UNIV, UNICAEN, BIOTARGEN, Caen, France; 3 Clinique Equine, Ecole Nationale Vétérinaire d’Alfort, UPEC, Maisons-Alfort, France; 4 NOVOTEC, ZAC du Chêne, Europarc, Bron, France; 5 CWD-VetLab, USC 957, BPLC, INRA, Ecole Nationale Vétérinaire d'Alfort, Maisons-Alfort, France; University College Dublin, School of Veterinary Medicine, IRELAND

## Abstract

Osteoarthritis is a common cause of pain and economic loss in both humans and horses. The horse is recognized as a suitable model for human osteoarthritis, because the thickness, structure, and mechanical properties of equine articular cartilage are highly comparable to those of humans. Although a number of equine experimental osteoarthritis models have been described in the literature, these cases generally involve the induction of osteoarthritis in just one joint of each animal. This approach necessitates the involvement of large numbers of horses to obtain reliable data and thus limits the use of this animal model, for both economic and ethical reasons. This study adapts an established equine model of post-traumatic osteoarthritis to induce osteoarthritis-associated lesions in all 4 fetlock joints of the same horse in order to reduce the number of animals involved and avoid individual variability, thus obtaining a more reliable method to evaluate treatment efficacy in future studies. The objectives are to assess the feasibility of the procedure, evaluate variability of the lesions according to interindividual and operated-limb position and describe the spontaneous evolution of osteoarthritis-associated pathological changes over a twelve-week period. The procedure was well tolerated by all 8 experimental horses and successfully induced mild osteoarthritis-associated changes in the four fetlock joints of each horse. Observations were carried out using clinical, radiographic, ultrasonographic, and magnetic resonance imaging methods as well as biochemical analyses of synovial fluid and postmortem microscopic and macroscopic evaluations of the joints. No significant differences were found in the progression of osteoarthritis-associated changes between horses or between the different limbs, with the exception of higher synovial effusion in hind fetlocks compared to front fetlocks and higher radiographic scores for left fetlocks compared to the right. This model thus appears to be a reliable means to evaluate the efficacy of new treatments in horses, and may be of interest for translational studies in human medicine.

## Introduction

Osteoarthritis (OA) is a painful joint disease that is clinically characterized by heat, pain, swelling and a decreased range of motion in affected joints. This pathology is defined as a disease of diarthrodial joints with variable degrees of articular cartilage destruction, subchondral bone sclerosis and marginal osteophyte formation [1]. Articular injuries of all types can lead to the development of OA. It is one of the most prevalent and debilitating diseases affecting both humans and horses, and has an extremely negative economic impact [2–4]. A U.S Department of Agriculture survey performed in horses indicates that up to 60% of lameness is related to OA [2]. The fetlock is the most commonly reported joint affected by traumatic and degenerative lesions in equine athletes [5]. Metacarpophalangeal joint disease has been reported as a major cause of lameness, lost training days and lost income in the thoroughbred racehorse industry. Another study showed that one third of 2- and 3-year-old thoroughbred horses had metacarpophalangeal cartilage lesions and OA [6].

Several of the experimental animal models described in the literature replicate symptoms of OA in order to develop early diagnosis methods or to study newly developed therapeutic strategies. Similarities between humans and horses in the pathogenesis, clinical presentation and pathological changes of OA are driving the development of translational studies in the horse [7,8]. Equine models provide many advantages including anatomical and histological similarities to human joints. Specifically, the horse has articular cartilage thickness, cellular structure, biochemical composition and mechanical properties that are highly comparable to those of humans. It is also easy to perform follow-up tests such as synovial fluid collection and imaging and obtain tissue samples from large animals. Nevertheless, the use of the horse as a model is limited in experimental studies due to the high management and housing costs and the greater ethical considerations compared to smaller animal models. Animal welfare would therefore be improved by reducing the number of animals involved in experimental OA studies. Most published experimental equine OA models [9–15] induce OA in just one joint of each animal, thus increasing the number of animals involved in these studies. In addition to the high costs and ethical concerns involved, this type of experimental design does not take individual variation of healing capacities into consideration. Indeed, we have frequently observed in our clinical practice that whilst some horses develop marked signs of OA, other horses are unlikely to do so. Similarly, some horses seem to have a better healing capacity than others.

Experimental osteochondral fragmentation models closely resemble the spontaneous OA process [16,17] and induce secondary signs of OA [11,12]. A previous 16-week study demonstrated that osteochondral fragmentation of the proximal phalanx alone provided clinical, imaging and histological signs of metacarpophalangeal joint OA and could be used to monitor the response to therapy [12]. Progressive lameness occurred when a similar procedure was combined with the creation of a full-thickness cartilage lesion on the dorsal aspect of the medial part of the metacarpal condyle, with local histological evidence of OA-associated changes that were not observed in an 11-week period study of sham-operated controlateral fetlock joints [18].

The present study was aimed to modify the model previously described [12] to develop an experimental design where OA is induced in the 4 fetlock joints of the same animal. Two main advantages were expected. The first was to overcome individual variability, using each horse as his own control instead of comparing a treated group with a control group. The second was to reduce the number of animals involved in tests in order to meet 3R ethical standards and to reduce the financial cost of equine studies. The objectives were 1) to assess the feasibility of performing osteochondral fragmentation of the proximal phalanx on all 4 fetlock joints of a horse; 2) to use clinical, imaging, biochemical and postmortem gross and histological parameters to evaluate the variability of the progression of induced lesions in terms of interindividual variability, left/right operated limb variability, and front/hind limb variability; 3) to describe the spontaneous evolution of OA-associated pathological changes over a 12-week period. We hypothesized that this experimental design would be well tolerated and that there would be no significant differences in the progression of induced lesions between horses or between operated limbs (front *vs* hind and left *vs* right). We also hypothesized that there would be an increase in monitored OA-associated changes during the 12-week study period in all OA-induced fetlock joints. This model was designed to provide a more reliable evaluation of treatment efficacy.

## Materials and methods

### Animals and study design

Eight clinically sound French Standardbred horses owned by the Center of Imaging and Research on the Equine Locomotor Affections (CIRALE) were included in the study. There were 4 geldings and 4 mares, median age was 3 years (ranging from 3 to 4) and median weight was 440.5 kg (ranging from 402 to 483 kg). All horses had been trained for racing before being retired due to a failure to trot at racing speeds. All were conditioned on treadmills before the beginning of the study.

**Fig 1:**
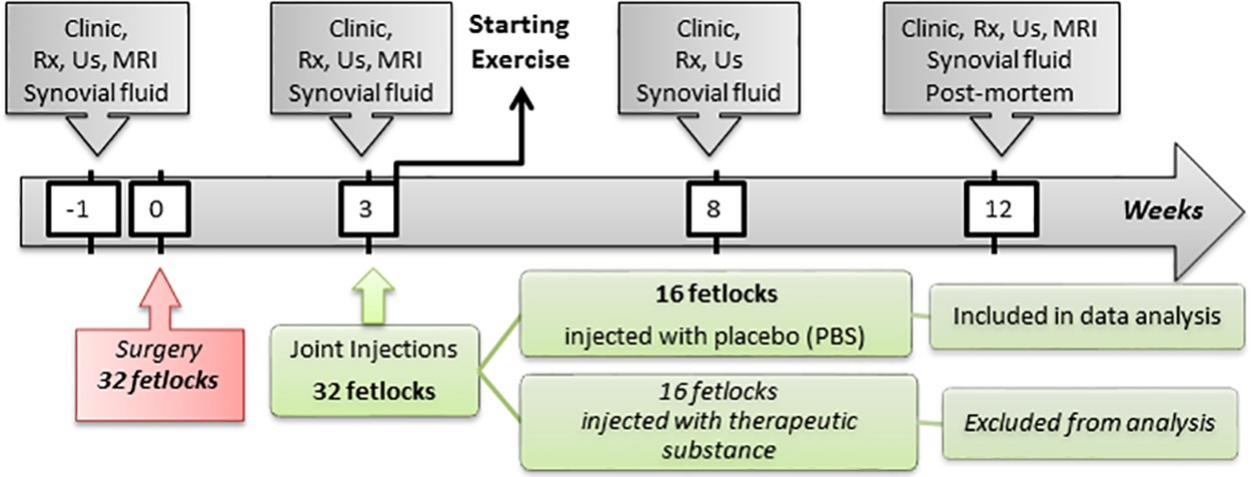
Diagram of the study timeline. Clinic—clinical evaluation; Rx—Radiographic examination; Us—Ultrasound examination; MRI—Magnetic resonance imaging; Synovial fluid—Synovial fluid sampling and analysis; Post-mortem—Post-mortem analysis including macroscopic and microscopic examination of the joints after euthanasia; PBS—phosphate-buffered saline.

The horses included in this study were part of a larger program evaluating the therapeutic effects of stem cell-based therapy on experimentally induced OA. Our study is based on data from the untreated or physiological saline placebo-treated joints, thus excluding data from the joints treated with stem cells (Fig 1). One week before the surgical induction of lesions (W-1), each horse was clinically evaluated for lameness and underwent full radiographic and ultrasonographic examination of its four fetlock joints to rule out the presence of pre-existing OA before being included in the study. Surgery was carried out on week 0. The first post-surgical follow-up examination was performed on week 3 (W3). After W3 follow-up examinations and samplings, one front fetlock and one hind fetlock of each horse were randomly selected by drawing lots to ensure a fair distribution of the number of right and left fetlocks, then injected with stem cell-based therapy. From this time onwards, the fetlocks injected with the stem cells were excluded from the current study, thus decreasing the number of monitored fetlocks to 16 for the last 2 examinations, performed on week 8 (W8) and week 12 (W12) (Fig 1). All 16 remaining fetlocks received a 2 mL intra-articular injection of phosphate-buffered saline (PBS) on W3. To avoid any joint flare reaction after intra-articular injections of the stem cells, all horses received one concomitant intravenous injection of nonsteroidal anti-inflammatory drugs with 1.1 mg/kg flunixin meglumine on W3. This protocol was approved by the ComEth Anses/ ENVA/ UPEC Ethical Committee (Permit number: 10/03/15-12). Surgery was performed under general anesthesia and every effort was made to minimize suffering and stress.

### Surgical technique

The procedure was performed by a board certified equine surgeon (CM), previously trained on cadaveric limbs, using an adaption of the protocol published by Boyce *et al*. (2013) [12]. This technique consisted of creating an osteochondral chip fragment in both metacarpophalangeal joints and both metatarsophalangeal joints of each horse (S1 Fig). Surgeries were performed on week 0 (W0) under isoflurane general anesthesia in dorsal recumbency with the 4 fetlocks aseptically prepared. An arthroscope was inserted through a dorsolateral portal. Before induction of the lesion, the dorsal aspect of each joint was assessed by arthroscopy for the presence of any pre-existing lesions. A proximo-medial instrument portal was then created. This portal allowed an 8 mm curved osteotome to be directed perpendicular to the articular surface of the proximal phalanx. The osteotome was struck with a mallet, creating a 2 mm thick osteochondral fragment of the proximo-dorso-medial border of the proximal phalanx. Contrary to the technique developed by Boyce *et al*. (2013), the resulting fragment was not repositioned in the fractured bed but the subchondral bone was exposed and then debrided using a straight curette to form a 15 mm-wide defect bed, as described for the carpal osteochondral fragmentation model [11] and the combined fragment-groove fetlock model [18]. The dorsal edge of the fragment remained attached to the joint capsule but was freed from the proximal phalanx. Debris from this procedure was not actively flushed in order to maximize joint inflammation, as previously reported [18]. Operated joints were then routinely closed and bandaged.

### Postoperative care and exercise program

All horses were confined to 3X4 m stalls for 3 weeks after surgery and heart rate, respiratory rate, body temperature and appetite were monitored twice daily to identify any signs of discomfort. Treatment was initiated prior to induction of anaesthesia, as follows: intramuscular procaine penicillin G injections for three days (22,000 IU/kg BID) and a six-day administration of phenylbutazone (2.2 mg/kg, intravenous every 12h for 2 days, then 2.2 mg/kg per os every 12h for 1 day and 1.1 mg/kg per os every 12h for 3 days). Bandages were changed every 2 days until suture removal fourteen days after surgery. Horses were turned out into small paddocks (10X10 m) on dry lots from the day after stem cell injection onwards.

Exercise was initiated progressively on W3 and consisted of working on a high speed treadmill for a 2-minute trot (5 m/s), 2-minute high speed trot (9 m/s) and 2-minute trot (5 m/s) 3 days a week, following conventional methods [11,12]. These days of treadmill exercise were alternated with 2 consecutive days of lunged trotting for 25 minutes, with equal amounts of right and left circles.

### Clinical assessment of the joint and investigation via imaging techniques

Clinical, radiographic, ultrasonographic and magnetic resonance imaging (MRI) evaluation of each fetlock were performed during W-1, W3, W8 and W12, although MRI was not performed on W8 (Fig 1). Clinical evaluations were performed by two equine locomotor pathology specialists (SJ and LB) and blind assessment of radiography, ultrasonography and MRI images was retrospectively carried out by two board-associated veterinary imaging specialists (FA and JMD). Scores were fixed by consensus agreement, during which parameters with different scores assigned by the observers were reviewed and discussed. A final score was then assigned by consensus of both observers.

Clinical evaluations were composed of the measurement of fetlock joint circumference and the evaluation of sensitivity to static digital flexion tests and joint effusion using a five-point scale ranging from normal to severe (0-normal, 1-mild, 2-moderate, 3-substantial, 4-severe), as previously described [19] (Table 1). As the study design did not permit the evaluation of bilateral lameness, follow-up lameness evaluations were only performed one day, three days and seven days after the injection of the stem cells (W3) to rule out the presence of an adverse event (i.e. a ≥ 3/5 lameness grade) causing a change in the weight bearing of the limbs and thus affecting the results of the present study. Degree of lameness was then graded on a scale of 0 to 5 in accordance with the lameness scale of the American Association of Equine Practitioners [20].

**Table 1:** Grading systems used for the clinical evaluation of sensitivity to digital flexion tests and fetlock joint effusion.

Score		Sensitivity to digital flexion tests	Fetlock joint effusion
0	Normal	No reaction of the horse to the flexion of the fetlock joint when moderate strength is used (3kg).	Concave appearance of the proximo-palmar recess of the metacarpo(tarso)phalangeal joint. No lateral swelling when medial pressure is applied to the recess.
1	Mild	The horse reacts to the flexion of the fetlock joint (withdrawal of the limb) when moderate strength is used (3kg).	Flat appearance of the proximo-palmar recess of the metacarpo(tarso)phalangeal joint. Mild lateral swelling when medial pressure is applied to the recess.
2	Moderate	The horse reacts to the flexion of the fetlock joint (withdrawal of the limb) when light strength is used (1kg).	Convex appearance of the proximo-palmar recess of the metacarpo(tarso)phalangeal joint. Lateral swelling easily obtained when medial pressure is applied to the recess.
3	Substantial	The horse reacts to the flexion of the fetlock joint (withdrawal of the limb) before strength is used.	Convex appearance of the proximo-palmar recess of the metacarpo(tarso)phalangeal joint beyond the suspensory ligament branches (third interosseous muscle). Soft consistency of the recess on palpation.
4	Severe	Violent withdrawal of the limb when flexion is applied to the fetlock joint without strength	Convex appearance of the proximo-palmar recess of the metacarpo(tarso)phalangeal joint beyond the suspensory ligament branches (third interosseous muscle) with a hard consistency of the recess on palpation, indicating synovial pressure. Synovial distension of the dorsal recess of the joint.

Radiographic examination was composed of lateromedial, dorsopalmar (20° proximodistal), dorsolateral-palmaromedial 35° oblique and dorsomedial-palmarolateral 35° oblique views of the 4 fetlock joints using digital radiography (Sound Eklin, Carlsbad, CA, United States). Osteophyte formation on the dorsal, lateral and medial aspects of the joint was scored according to osteophyte size (0 = none, 1 = small, 2 = medium, 3 = large), as described in the literature [21,22]. A maximum cumulative score of 9 could be obtained.

A 6 MHz linear transducer (Hitachi Medical Systems, Saint-Priest, France) was used to carry out transverse and longitudinal ultrasound scans on the dorsal and collateral aspects of each fetlock, following the scanning technique previously described [23]. Osteophyte formation on the dorsal, lateral and medial aspects of the joint was graded following the same criteria used for radiographic evaluation. Synovial fluid effusion was also graded in ultrasound images of the palmaro/plantaro-proximal recess of the joint using a similar 5-point scale to that used for the clinical evaluation (Table 2).

**Table 2:** Grading system used for ultrasound evaluation of synovial fluid effusion of the fetlock joints.

Score		Synovial fluid effusion
0	Normal	Small amount of fluid in the proximo-palmar recess of the metacapo (tarso) phalangeal joint. Concave appearance of the skin. No motion of the fluid when the recess is pressed.
1	Mild	Small amount of fluid in the proximo-palmar recess of the metacapo (tarso) phalangeal joint. Concave to flat appearance of the skin. Motion of the fluid when the recess is pressed.
2	Moderate	Moderate amount of fluid in the proximo-palmar recess of the metacapo (tarso) phalangeal joint. Convex appearance of the skin. Motion of the fluid when the recess is pressed.
3	Substantial	Substantial amount of fluid in the proximo-palmar recess of the metacapo(tarso)phalangeal joint and small amount of fluid in the dorsal recess of the joint. Convex appearance of the skin.
4	Severe	Large amount of fluid in the proximo-palmar recess of the metacapo(tarso)phalangeal joint and substantial amount of fluid on the dorsal recess of the joint with synovial pressure. Convex appearance of the skin.

MRI was performed with horses standing under sedation. Low field strength 0.27 Tesla Magnet and a fetlock receive coil (Hallmarq Veterinary Imaging, Guildford, Surrey, UK) were used. Images were acquired in transverse, dorsal and sagittal planes using T1-weighted gradient echo, T2-weighted fast spin echo (FSE), and Short Tau Inversion Recovery (STIR) FSE sequences with motion correction software (MI) for FSE sequences (S1 Table). The images obtained were then graded using a previously described semi-quantitative scoring system [24] adapted for use in standing equine fetlock joint MRI. Both proximal phalanx and metacarpal/tarsal condyle were assessed for the presence of oedema-like bone marrow lesions (0–3), subchondral bone sclerosis (0–3) and osteophyte formation (0–3). Soft tissue structures associated to the metacarpo/tarsophalangeal joints were also graded for synovial fluid effusion (0–3), synovial membrane thickening (0–3), joint capsule oedema (0–3) and joint capsule thickening (0–3). A maximum cumulative score of 27 could be obtained.

### Synovial fluid analysis

Synovial fluid (1 mL) was sampled from each joint on W-1, W3, W8 and W12 (Fig 1). Sampling was performed under sedation (intravenous administration of a combination of detomidine 0.01 mg/kg and butorphanol 0.01 mg/kg) after aseptic preparation of the skin. Using a lateral approach on the flexed limb, a 20-gauge needle was inserted between the metacarpal/metatarsal condyle and the lateral proximal sesamoid bone. Approximately 0.3 mL of fluid was placed in an EDTA tube and the total nucleated cell count was directly measured using an automatic analysis system (Sysmex XN10, Sysmex Corporation, Japan) (cells/μL). The remaining fluid was placed in a dry tube for centrifugation at 2500 × g for 10 minutes. After aqueous phase recovery, 20 μL of the sample was placed on a refractometer (Zuzi series 300, Auxilab SL, Spain) to determine the total protein concentration. The remainder was then stored at -80° C for ELISA analysis. Synovial fluid concentration of prostaglandin E_2_ (PGE_2_) and C-terminal telopeptide of type II collagen (CTX-II) were estimated as markers of inflammation and cartilage turnover, respectively, through the use of commercially available high-sensitivity enzyme immunoassay kits (Prostaglandin E_2_ Parameter Assay Kit, R&D systems, USA; Serum Pre-Clinical CartiLaps^®^ (CTX-II) EIA, Immunodiagnostic Systems Holdings PLC, UK) [25,26].

### Postmortem examination

Twelve weeks after surgery (W12), all horses were euthanized with an intravenous injection of a mixture of 1g embutramide, 2.5g mebezonium and 250mg tetracaine. Both metacarpophalangeal and both metatarsophalangeal joints of each horse were opened dorsally to expose the articular surfaces. Articular surfaces were photographed and changes in metacarpal/tarsal condyles were blindly scored according to the Osteoarthritis Research Society International (OARSI) Guidelines [27] by two investigators (LB and SJ) for wear-lines, erosions and osteochondral lesions using a 4-point scale ranging from 0 to 3. A maximum cumulative score of 9 could be obtained.

Osteochondral sections were harvested from the medial part of the distal aspect of the metacarpal/tarsal condyle and processed for histological and immunohistological analysis (S2 Fig). Samples were fixed with neutral buffered formalin (Carlo Erba Reagents) and embedded in paraffin. After fixation, 5 mm latero-medial slices were cut and mounted on coated glass slides. The paraffin was removed and the antigenic sites were unmasked by treatment with 0.5% hyaluronidase. Sections were incubated overnight at 4°C with rabbit anti-human type I collagen primary antibody (Novotec, 20111) or rabbit anti-human type II collagen (Novotec, 20211). After inhibition of the endogenous peroxidases by hydrogen peroxide, the sections were incubated in a peroxidase-conjugated secondary antibody (Dako, Envision rabbit, ref. K4002). The reaction with diaminobenzidine substrate (Dako, K3468) reveals the antigen-antibody complexes through the appearance of brown staining. The sections were counter-stained with Mayer hematoxylin then mounted between the slide and coverslip in an aqueous medium. The primary antibody was replaced by 3% PBS—diaminobenzidine as a negative control.

Slides were observed with an optical microscope (Leica DM2000) connected to a digital camera (Leica DFC420C) driven by image acquisition software (LAS V4.2). The images obtained were retained and selected for the production of photo plates using image processing software (Adobe, Photoshop CC2015.5). Lesions were blindly scored according to the OARSI histopathology evaluation system [28] by two independent investigators (DJH and MM). This evaluation system took into account the severity of cartilage and bone lesions (grade 0–6), the proportion of affected area in relation to the total area (grade 0–4), and the lesion depth (grade 0–4), allowing a more precise differentiation of the lesions. The total histological score of the lesions was the multiplication of these 3 parameters, with a maximum possible score of 96.

### Statistical analysis

Statistical analysis was performed after the data had been visually assessed for normality. The variables were first presented descriptively: the mean and standard deviation of the normally distributed continuous outcome (fetlock joint circumference) and the median and quartiles of the other continuous outcomes were calculated at each time point for each fetlock group, while the number of observations contained within each grade and the median were calculated for categorical outcomes, Fetlocks injected on W3 with the stem cell-based therapy were excluded from further statistical analyses, leaving a total of 16 fetlocks for statistical analysis.

The comparison of interindividual variability and operated limb variability (left *vs* right and front *vs* hind) was carried out by analyzing the values resulting from the differences between W12 and W-1 scores, excluding the post-mortem scores. The differences between W12 and W-1 values of synovial fluid biomarkers were normally distributed and had equal variance, as revealed by the Levene test. Other differences were not normally distributed. Interindividual variability was evaluated by a one-way analysis of variance for normally distributed outcomes and by a Kruskal-Wallis test for all other outcomes. Similarly, Student’s *t*-test or the nonparametric Mann-Whitney procedure were used to test for differences between fore- and hind-fetlocks and between left and right fetlocks.

The comparison between W-1 values as the pre-osteoarthritis baseline, and between W12 values as the post-osteoarthritis state was also analyzed with 2 different tests depending on the variables studied. The non-parametric Wilcoxon signed-rank test was performed when the outcome was not normally distributed, while paired Student’s *t*-tests were used when the outcome was normally distributed. The paired W-1 and W12 values of each fetlock were then compared with these tests.

All statistical analyses were performed using R software (version 3.4.3; R Foundation for Statistical Computing, Vienna, Austria, 2017) or Excel 2016 for Windows (Microsoft, Redmond, DC, USA). A p-value of 0.05 was considered to be significant.

## Results

### Surgical induction of the lesions and postoperative follow-up

Osteochondral fragmentation was successfully performed on all 32 fetlocks. The median duration of surgery was 1 hour and 5 minutes (1:00–1:12), with 2 hours and 9 minutes of anaesthesia (2:00–2:40). An average of 1 hour (0:48–1:06) was needed for the horses to stand up following anaesthesia.

Body temperature, heart rate, respiratory rate and appetite remained normal throughout the study period for all individuals except for one, which showed elevation of its heart and respiratory rates 12 hours after surgery, associated with signs of colic (pawing at the ground). The intravenous administration of 25 mg/kg dypirone resolved these clinical signs within 4 hours. No horse showed evidence of heat, pain or swelling of the metacarpophalangeal and metatarsophalangeal joints when bandages were changed. Horses moved comfortably in their box and paddocks and no behavioural evidence of pain was noted. No adverse events were observed after the injection of stem cell-based therapy in W3 (S1 Dataset).

### Variability in lesion evolution

Horses were homogeneous in terms of age, breed, weight and activity. Interindividual statistical analyses did not reveal any significant difference between horses when considering the differences between W12 and W-1 scores of clinical and imaging parameters and the differences between W12 and W-1 values of synovial fluid parameters. Similarly, no significant interindividual differences were observed in the distribution of macroscopic and microscopic scores by the end of the study (S2 Dataset and S2 Table).

**Fig 2:**
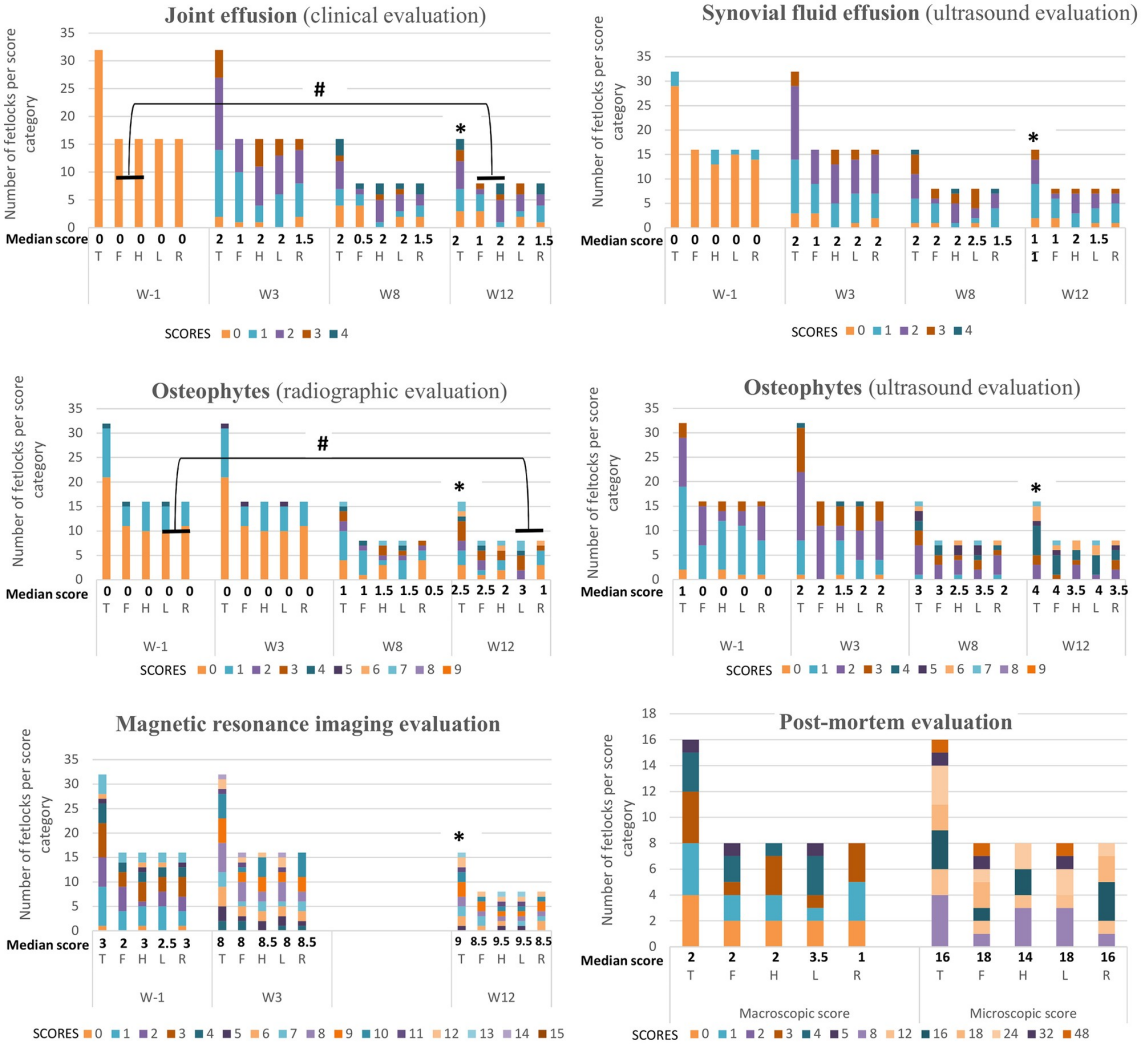
The evolution of the median scores and of the number of fetlocks per score category and per position (T, F, H, L, R) over the study period (W, weeks) for 32 fetlocks on W-1 and W3 and for 16 fetlocks on W8 and W12. Post-mortem scores were measured on W12. T—Total fetlocks, *i*.*e*. all fetlocks pooled together; F—Fore fetlocks, *i*.*e*. all fore fetlocks pooled together; H–Hind fetlocks *i*.*e*. all hind fetlocks pooled together; L–Left fetlocks, *i*.*e*. all left fetlocks pooled together; R—Right fetlocks, *i*.*e*. all right fetlocks pooled together. # Significant difference between the two fetlocks positions considering the difference between W12 and W-1 values with p<0.05. * Significant difference between paired values of the same fetlocks for that time point compared to W-1 with p<0.05.

In addition, statistical analyses did not reveal any significant differences between left and right fetlocks, with the exception of a radiographic score with higher values for left fetlocks than for right fetlocks (Fig 2 and S3 Table). Although fore and hind fetlocks were not significantly different for most of the parameters, hind fetlocks produced significantly more synovial effusion after surgery than front fetlocks (Fig 2 and S3 Table).

### Clinical follow-up

**Fig 3:**
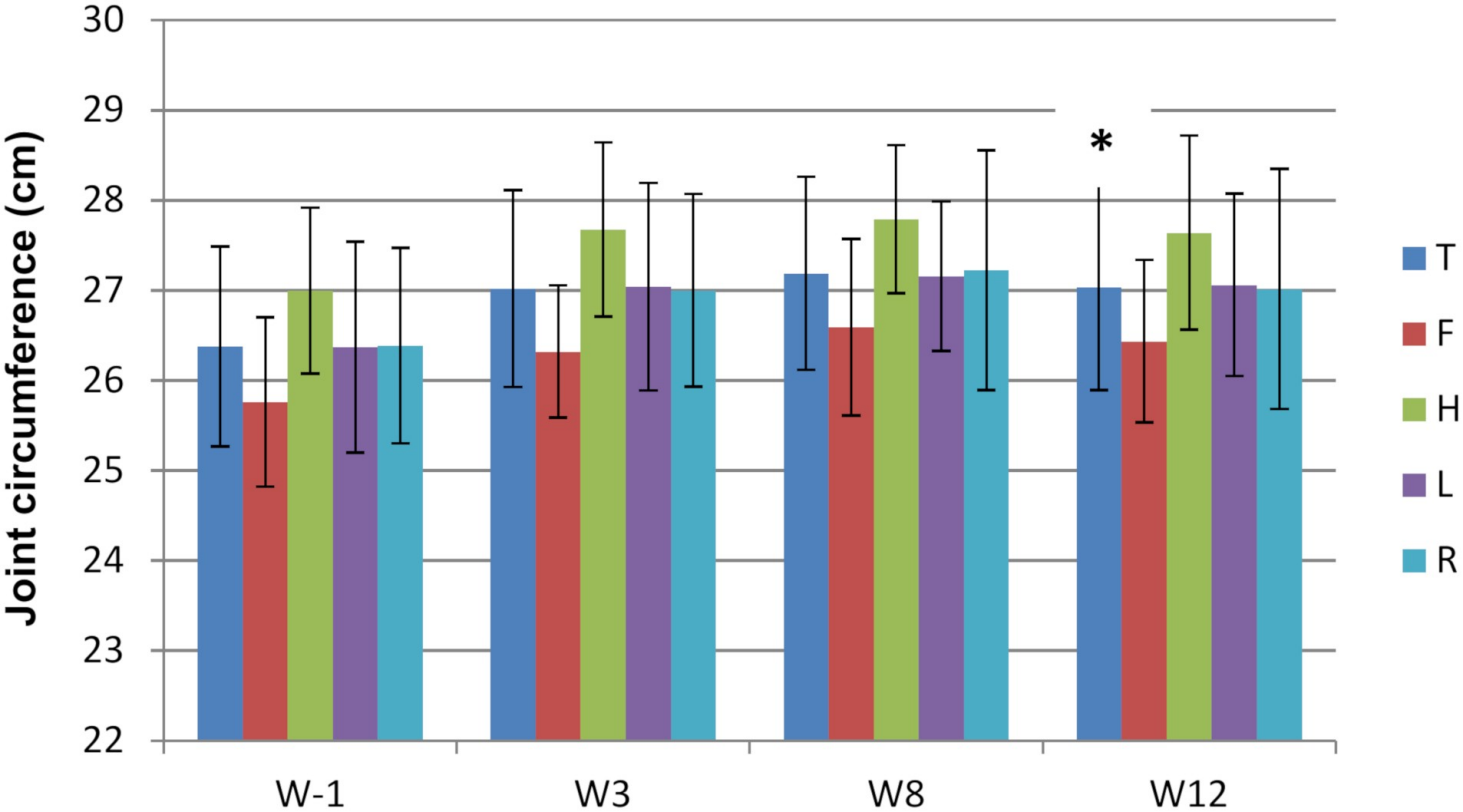
Evolution of the mean (standard deviation) fetlock joint circumference over the study period measured in 32 fetlocks on weeks -1 (W-1) and 3 (W3) and in 16 fetlocks on weeks 8 (W8) and 12 (W12) and displayed per fetlock position. T—Total fetlocks; F—Fore fetlocks; H—Hind fetlocks; L—Left fetlocks; R–Right fetlocks. * Significant difference between paired values of the same fetlocks for that time point compared to W-1 with p<0.05.

None of the 8 horses showed sensitivity to digital flexion tests throughout the study period (S2 Dataset). Ninety-four percent (30/32) of the fetlocks showed an increased joint effusion score on W3 compared to baseline (S2 Dataset and Fig 2). The joint effusion score and the fetlock joint circumference were significantly higher in W12 than in W-1 when comparing the paired values of each fetlock (Figs 2 and 3).

### Follow-up with imaging techniques

At the end of the study (W12), imaging signs of OA were mild to moderate. Indeed, whatever the imaging parameter, the scores reached on W12 were below 50% of the maximal attainable grading score for 11/16 fetlocks (69%). None of the fetlocks had high MRI severity scores (score ≥ 18/27), and only 4/16 fetlocks (25%) had high radiographic and ultrasonographic severity scores (score ≥ 6/9) (S2 Dataset and Fig 2).

**Fig 4:**
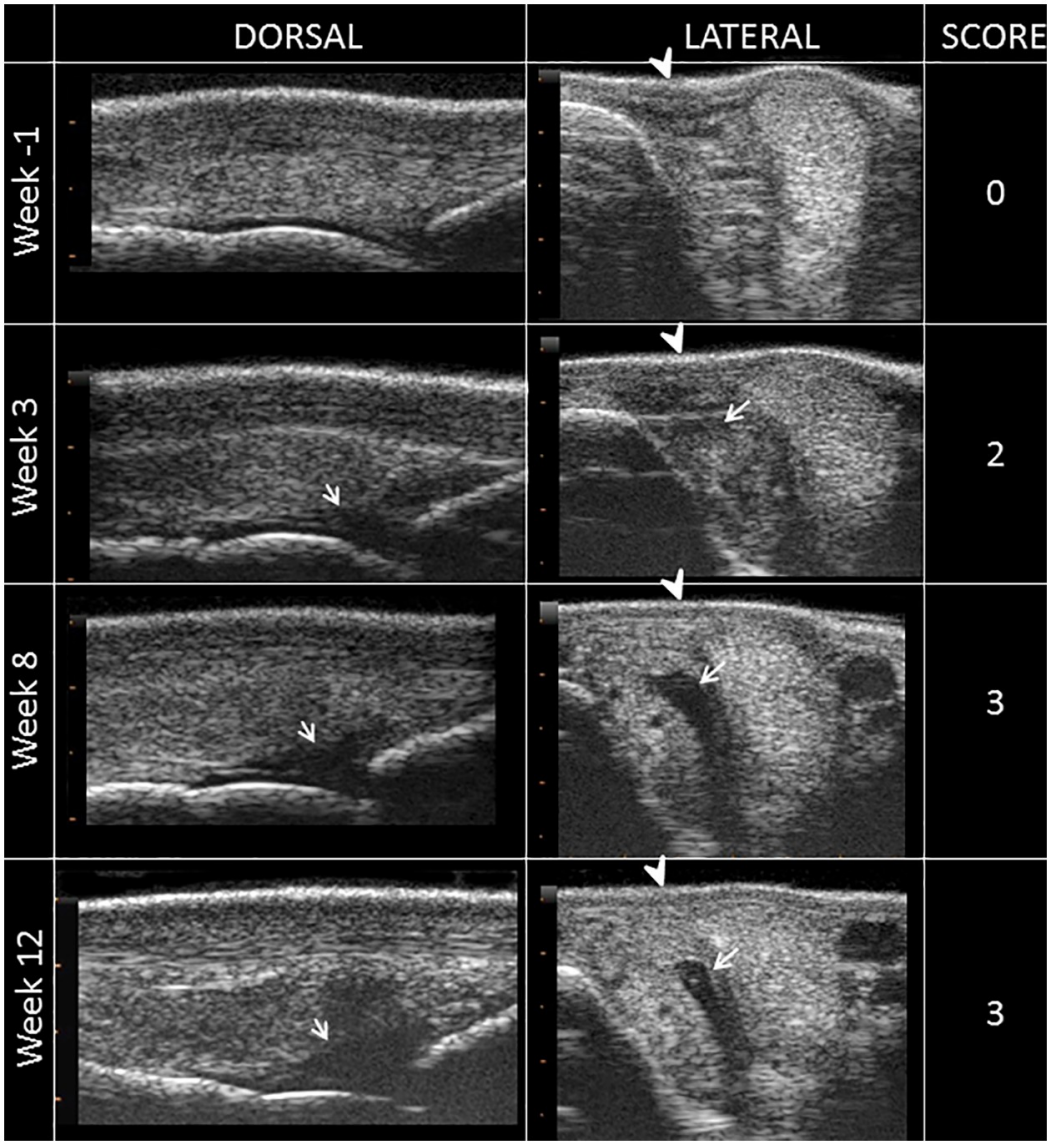
Inclusion and follow-up ultrasound sections of the left metacarpophalangeal joint of horse 7. A longitudinal section of the dorsal aspect (DORSAL) and a transverse section of the lateral aspect (LATERAL) of the joint show the different grades of synovial effusion measured on ultrasound. Note the evolution of the skin appearance (arrow head) from concave to convex and the increase in the amount of fluid (arrow) from moderate to substantial.

**Fig 5:**
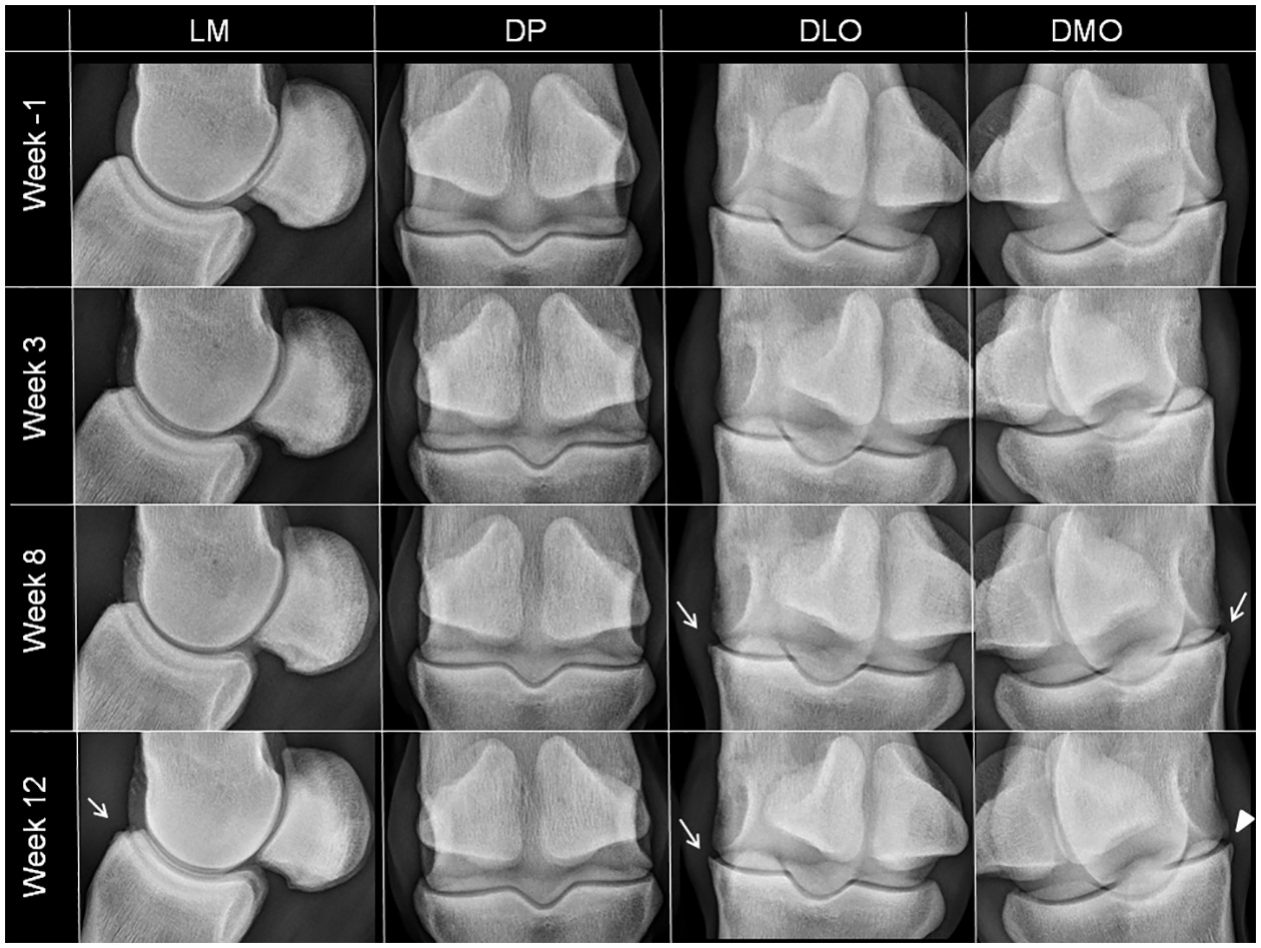
Inclusion and follow-up radiographic views of the left metacarpophalangeal joint of horse 7. There is a development of grade 2 (white arrows) and grade 3 (white triangle) osteophytes. LM—latero-medial views; DP—dorso-palmar views, DLO—dorsolateral-palmaromedial 35° oblique views; DMO—dorsomedial-palmarolateral 35° oblique views.

**Fig 6:**
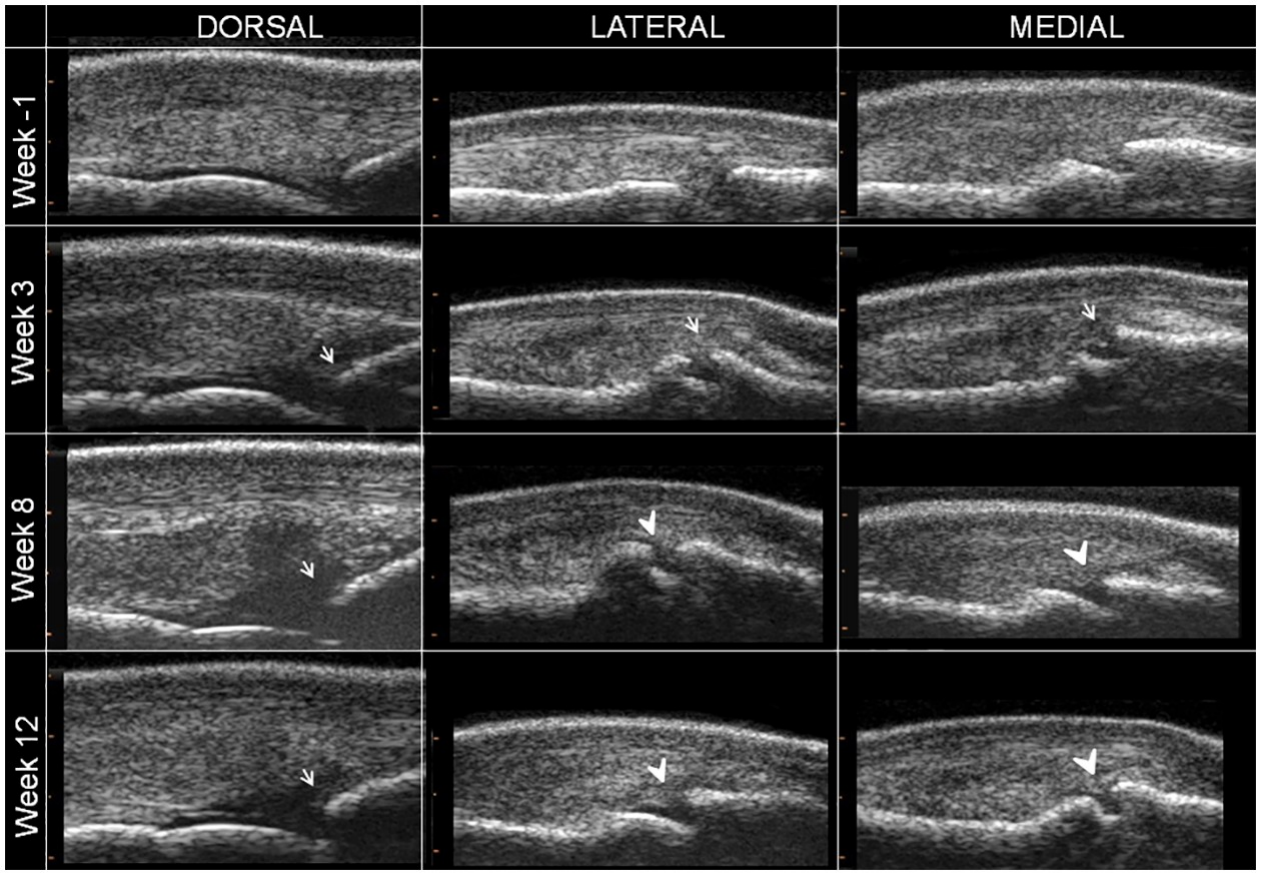
Inclusion and follow-up longitudinal ultrasound sections of the left metacarpophalangeal joint of horse 7. Sections of the dorsal aspect (DORSAL), dorso-lateral aspect (LATERAL) and dorso-medial aspect (MEDIAL) of the joint reveal a development of grade 1 (white arrows) to grade 3 (white arrow head) osteophytes.

**Fig 7:**
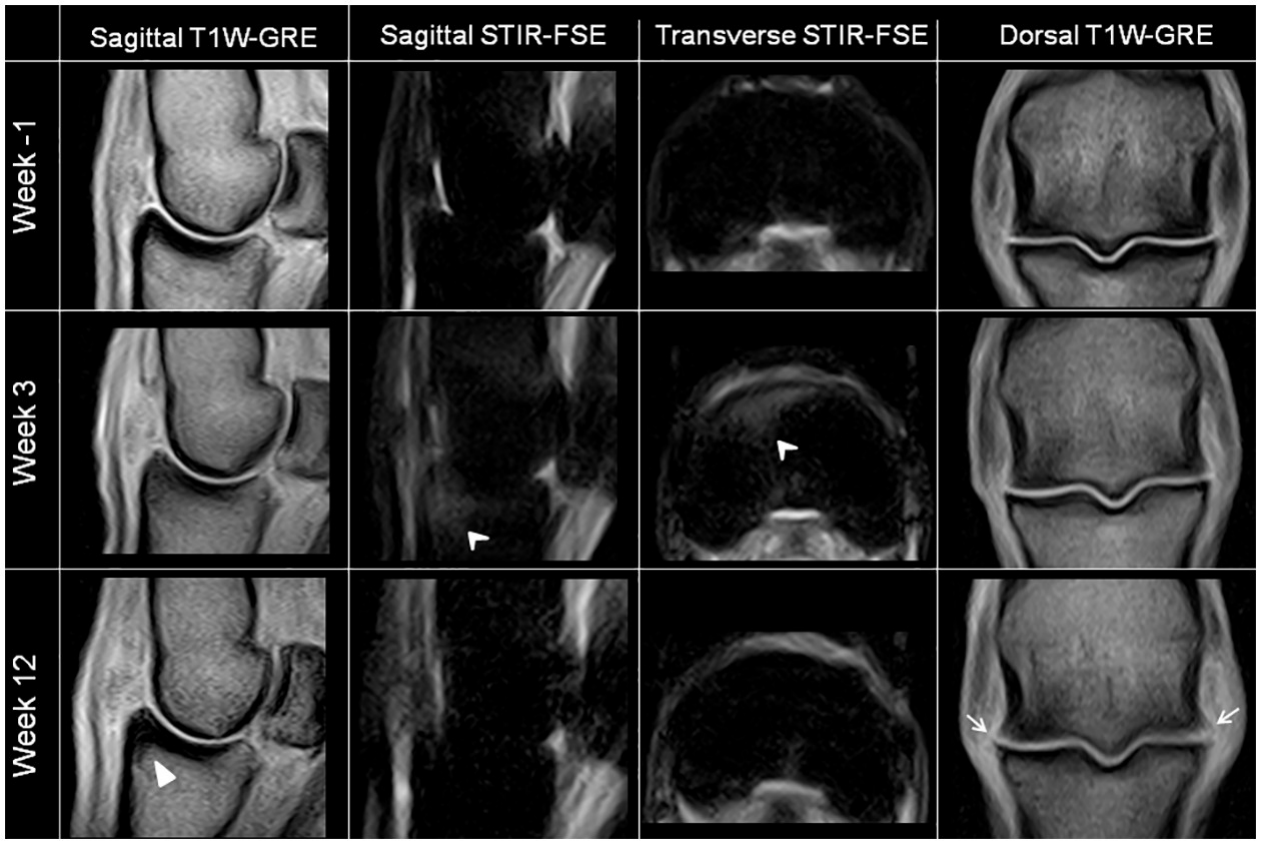
MRI images of the left metatarso-phalangeal joint of horse 5 (sagittal and transverse images) and of the left metacarpo-phalangeal joint of horse 7 (dorsal images) obtained successively at weeks -1 (inclusion), 3 (post-fragmentation) and 12 (end of the study), showing the temporal progression of the OA-associated structural changes. There is evidence of grade 3 bone marrow oedema-like lesions (arrow heads) and grade 1 bone sclerosis (white triangle) on the proximal phalanx of the left metatarsophalangeal joint of horse 5 near the fragmentation site. Also note the grade 3 periarticular osteophytes (white arrows) on the left metacarpophalangeal joint of horse 7. T1W GRE—T1 weighted ultra-fast gradient echo sequences; STIR-FSE—short tau inversion recovery fast spin echo sequences.

The effusion score measured by ultrasound reflected the results of the clinical follow-up, with a significantly higher score recorded in W12 than in W-1 (Figs 2 and 4). In addition, there was a progressive increase in osteophyte formation scores (for both radiographic and ultrasonographic evaluation) throughout the study period (Figs 2, 5 and 6, S3 Table and S2 Dataset), with a significant increase in scores on W12 compared to those of W-1 (Fig 2). The total MRI score was also significantly higher on W12 compared to W-1 (Fig 2). Not all MRI-measured parameters evolved in the same way along the study period (Table 3), in particular bone marrow edema-like lesions seemed to be mostly detected on the proximal phalanx on W3 (Table 3) and particularly around the osteochondral fragment (Fig 7), while little evolution occurred for subchondral bone sclerosis over the study period (Fig 7 and Table 3).

**Table 3:** MRI grading system and median scores obtained (first quartile- third quartile).

MRI criteria	W-1	W3	W12
Synovial fluid effusion (/3)	0 (0–1)	1 (1–1)	2.5 (1.75–3)
Synovial membrane thickening (/3)	0 (0–0)	0 (0–0)	0 (0–1)
Joint capsule oedema (/3)	0 (0–0)	1 (1–2)	1 (0–1)
Joint capsule thickening (/3)	0 (0–0)	1 (0–1)	1 (1–1)
Metacarpal/tarsal subchondral bone sclerosis (/3)	1 (0–1)	1 (0–1)	1 (0–1.75)
Proximal phalanx subchondral bone sclerosis (/3)	0 (0–1)	0 (0–1)	0,5 (0–1)
Metacarpal/tarsal oedema-like lesions (/3)	0 (0–0)	0,5 (0–1)	0 (0–1)
Proximal phalanx oedema-like lesions (/3)	0 (0–0)	2 (1–2.75)	1 (0–1)
Osteophyte formation (/3)	0 (0–1)	0 (0–1)	0,5 (0–2)
**TOTAL MRI SCORE (/27)**	**3 (1–4)**	**8 (6–9.75)**	**9 (7–10)**

### Evolution of synovial fluid parameters

**Fig 8:**
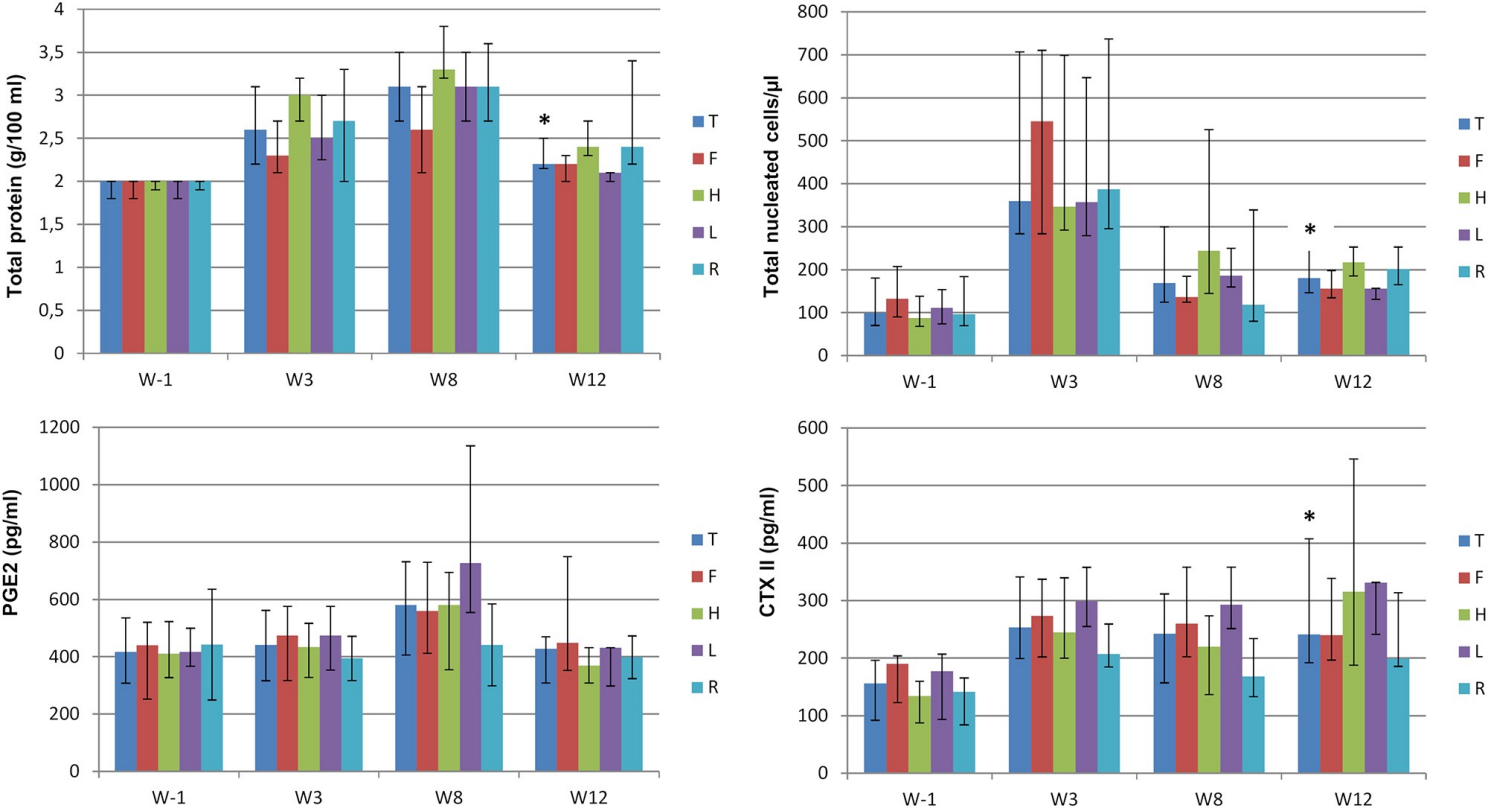
Evolution over the study period of the median values (1^st^ quartile- 3^rd^ quartile) of synovial fluid parameters measured in 32 fetlocks on weeks -1 (W-1) and 3 (W3) and in 16 fetlocks on weeks 8 (W8) and 12 (W12), also displayed by fetlock position. T—Total fetlocks; F—Fore fetlocks; H—Hind fetlocks; L—Left fetlocks; R—Right fetlocks. * Significant difference between paired values of the same fetlocks for that time point compared to W-1 with p<0.05.

A significant increase for total protein concentration, total nucleated cell counts and CTX-II concentration was observed between W-1 and W12 when comparing the paired values of each fetlock at that time points (Fig 8). Total protein concentration and total nucleated cell counts seemed to be at their highest on W3 and W8 (Fig 8, S3 Table and S2 Dataset) but no statistical comparisons were made at these stages. PGE_2_ variation was not significant.

### Post-mortem evaluation

**Fig 9:**
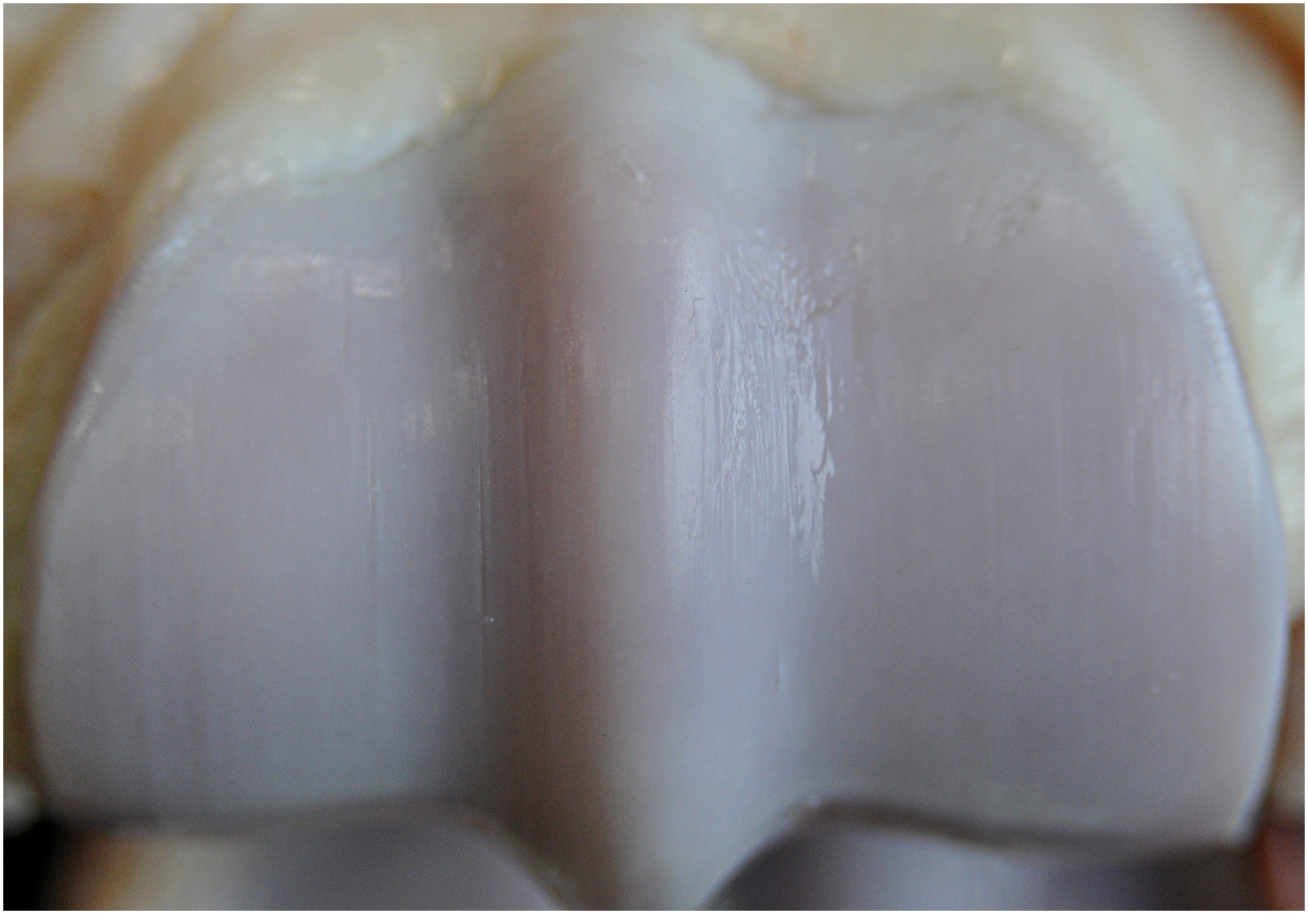
Gross image of the distal aspect of the right third metacarpal bone of horse 6. There are spontaneous OA-associated changes of the metacarpophalangeal joint, including grade 3 wear lines.

**Fig 10:**
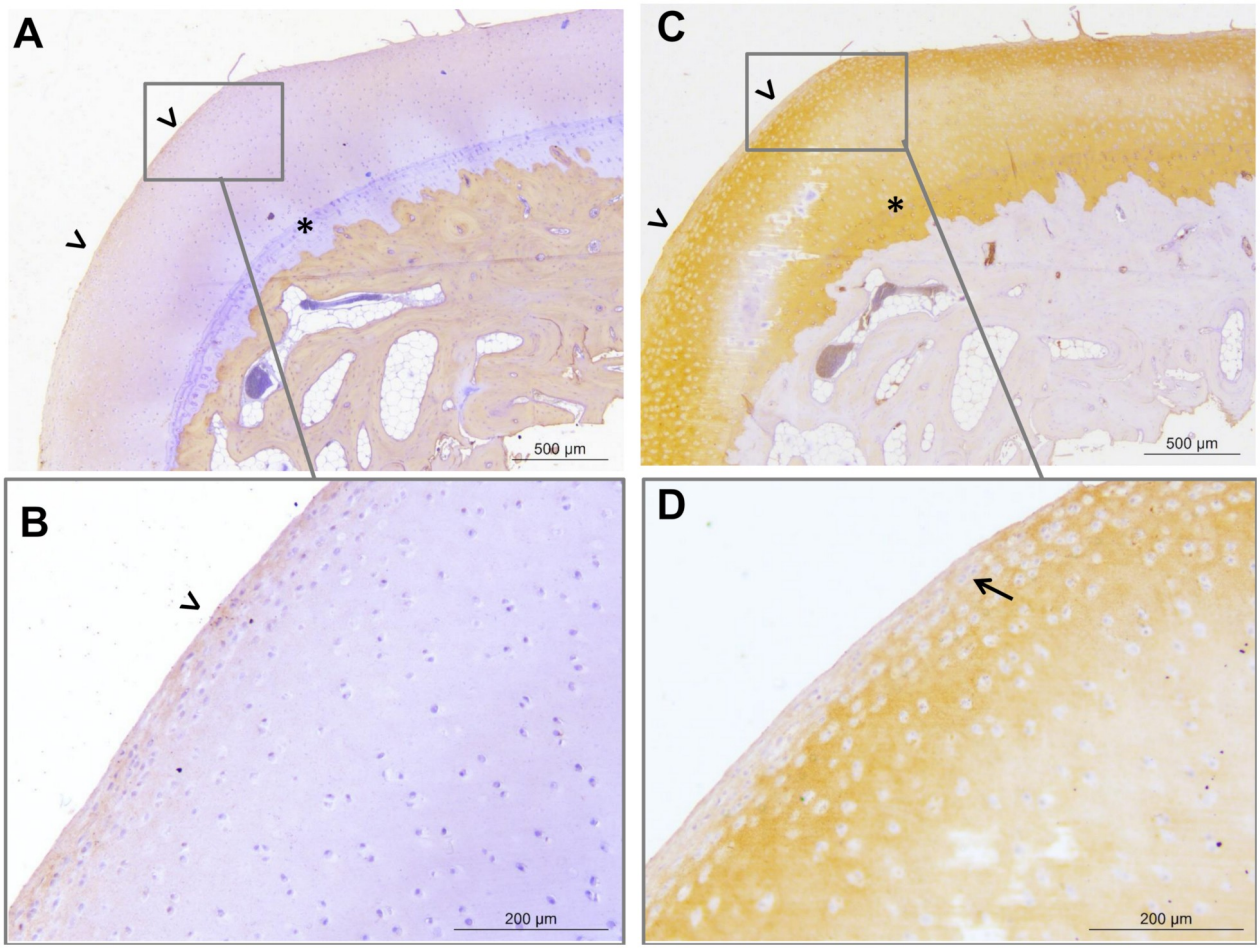
Representative light micrographs of osteochondral sections from the medial part of the distal aspect of the right metatarsal condyle of horse 8. Micrographs obtained on Week 12 illustrating a grade 16 histological score and revealing type I (A,B) and type II (C,D) collagen labelling (brown marking). Note the presence of a fibrocellular tissue (open arrow heads) that is partly positive for type I collagen and type II collagen. The superficial and intermediate zones of the articular cartilage are eroded, with fibrillations observed across the sample. Clusters of chondrocytes are observed near the fibrocellular tissue (arrows). The tidemark is visible (star).

All fetlocks showed macroscopic lesion scores below 55% of maximum values (≤ 5/9), with a majority (12/16 fetlocks) of low to moderate severity scores (≤ 3/9) (S2 Dataset). Most of the lesions observed were wear lines (Fig 9 and Table 4). On histological evaluation, all samples had values below 50% of the maximum lesion score (≤ 48/96), with a majority showing a low severity score of between 8 and 18 (11/16 samples) (S2 Dataset). No sample had a score < 8 and only one sample showed more severe lesions (a score of 48). The last 4 samples had an intermediate score of between 24 and 32 (Table 4 and Fig 2). Most of the samples were characterized by varying degrees of erosion of the superficial zone of cartilage and by the presence of a peripheral fibrocellular tissue (Fig 10) which had replaced the superficial and intermediate zones, or even the deep zone in some samples. Immunohistological analyses of this fibrocellular tissue revealed that these changes were associated with the presence of type I collagen matrix, with or without type II collagen, whilst healthy cartilage is characterized by type II collagen extracellular matrix. In addition, cytoplasmic collagen labeling corresponding to chondrocyte cellular activity was sometimes observed in the lesions. Changes in chondrocyte organization were also observed, with the presence of small clusters (Fig 10). When changes in cartilage structure were significant, small bone marrow vacuoles were noted near the mineralized cartilage. In addition, the tidemark became less visible or absent.

**Table 4:** Median macroscopic and microscopic scores obtained (first quartile—Third quartile) ^a^.

Median Macroscopic Score (Q1—Q3)		Median Microscopic Score (Q1—Q3)	
Wear lines (/3)	1 (0.75–2.5)	Severity of the lesion (/6)	2 (2–3)
Erosions (/3)	0 (0–1)	Extension of the lesion (/4)	3 (2–3)
Palmar/plantar osteochondral lesions (/3)	0 (0–0)	Depth of the lesion (/4)	2 (2–3.25)
**TOTAL (/9)**	**1.75 (0.75–3.5)**	**TOTAL (/96)**	**16 (11–24)**

^a^ The total macroscopic score was obtained by addition of the 3 parameters (3+3+3), while the total microscopic score was obtained by multiplication (6*4*4).

## Discussion

Surgical fragmentation performed on the proximal phalanx on both metacarpophalangeal and both metatarsophalangeal joints was well tolerated and successfully created mild OA-associated changes. These changes were significant for most of the measured parameters, indicating that mild changes should be sufficient to reliably evaluate the efficacy of a treatment. No significant difference was observed in the evolution of changes between horses or between fetlock positions (left, right, front or hind) for most of the outcome variables. The significant differences observed for certain outcomes do not preclude the use of this model in future studies. The antero-posterior difference found for synovial fluid effusion can be easily handled in efficacy studies by comparing each fetlock to its contralateral. The left to right difference found for the evolution of the radiographic score, i.e. a stronger progression in left fetlocks, was unexpected and may be explained by the small sample size. Horses had identical exercise lunging on the left and right rein or on a straight line on the treadmill. Compared to previously reported models of OA [9–12,15,18], the procedure described here thus has the advantage of reducing the number of horses involved in OA studies, as required by 3R ethical guidelines, by maximizing the number of joints that could be studied and compared per animal. This model is also advantageous for use in efficacy studies of therapeutic substances as it provides the opportunity to compare the evolution of OA-associated changes within the same individual.

The protocol used was more traumatic than the non-terminal protocol used in a previously published study [12]. First, modifications were made to the historical surgical technique, with two major changes: (1) the fragment in the fractured bed was not placed back in position, and the osteochondral lesion was burred back; (2) tissue debris was not flushed from the joint in order to maximize joint inflammation. These changes in the surgical technique were similar to those adopted in a recently published model study [18]. Second, the exercise program was a little different to that described in previous publications as it included lunge activity along with high-speed treadmill exercise. Additional lunging was chosen to enhance the development of OA-associated changes. Indeed, asymmetric bearing and misalignment between the hoof and the limb during curved motion induces collateromotion and axial rotation. Considering that the shapes of the distal joints are designed to undergo primarily flexion/extension movement, these asymmetrical movements contribute to joint surface damage [29]. Finally, the study duration was of 12 weeks here rather than the 16-week period reported in other studies. Although more obvious signs of OA would have been observed over a 16-week study, the results of this study confirm that a 12-week period is sufficient to develop progressive signs of OA.

All horses showed a mild response to the surgical procedure but the protocol described in this study was well tolerated, as confirmed by the follow-up of clinical parameters. Articular lesions induced by the combination of surgical procedure and exercise were always mild to moderate after 12 weeks for all outcomes, as previously reported [12]. Similar OA lesions are frequently encountered in clinical cases of horses with regular sport or racing activities, and do not exclude these horses from participating in the exercise program.

The use of imaging techniques is essential to confirm diagnosis of degenerative joint disease and evaluate the severity of the lesions. Radiography still provides an excellent representation of bones. Radiographic OA diagnosis is based on the presence of lysis or sclerosis of the subchondral bone, periarticular osteophytes and in some cases thinning of the joint space [30]. We retained periarticular osteophytes for use in the scoring system, because this radiographic sign was considered to be the least prone to measurement errors that could have occurred due to subtle changes in the radiographic contrast or potential differences in the magnification effect between successive examinations. Ultrasonography was used as a complementary tool. This technique has been reported to be more sensitive than radiography for the detection of periarticular irregularities, remodelling or osteophytes [31]. As ultrasonography has also been proven to be a very useful technique for the identification and description of dorsal and abaxial soft tissues of the fetlock in the horse [23], it was also used after the physical examination to document the presence of synovitis, which is another sign of OA. To our knowledge, this is the first study to monitor experimental lesions of OA using ultrasound in the horse. This technique played an interesting role in the follow-up of the joints, with scores increasing during the study for all fetlocks. The use of this technique can therefore be considered for future studies.

Nevertheless, radiography and ultrasonography do have limitations. Both lack the ability to precisely evaluate articular cartilage and subchondral bone. Computed tomography and MRI are imaging techniques that could overcome these limitations to provide a cross-sectional and three-dimensional evaluation [32]. High-field MRI performed under general anesthesia has already been proven to be useful in the temporal assessment of OA-associated changes occurring in the osteochondral fragmentation model of the equine middle carpal joint [33]. To our knowledge, this is the first temporal assessment of experimental lesions of OA through the MRI examination of standing horses. A combination of the most commonly clinically used sequences on the standing horse was chosen to perform this study in transverse, dorsal and sagittal planes. MRI was particularly useful for the identification of bone lesions with T2-weighted and fat saturation sequences highlighting the high fluid signal that is consistent with bone marrow oedema-like lesions, an important feature of articular injuries. Bone marrow oedema-like lesions were more frequent and severe on the images obtained during W3 (Table 3) and particularly around the osteochondral fragment (Fig 7), as previously reported [33]. These lesions, observed during W3, are more likely to reflect the presence of vascular (hemorrhagic) changes linked to the surgical injury [34]. Such injuries to the subchondral bone are suspected to predispose joints to the development of OA [35]. In addition, MRI is sensitive to changes in bone density and thus permits the diagnosis of subchondral bone sclerosis, another feature of OA. The study duration may have been too short to evaluate the development of subchondral bone sclerosis, because little evolution of scores was observed except for the area adjacent to the osteochondral fragmentation on the proximal phalanx (Fig 7). This result is similar to the changes reported in the previous study [33], where low-signal bone lesions developed in areas of previous high signal bone lesions. Finally, MRI provides detailed information about the alteration of bone surfaces for the detection of periarticular osteophytes [30]. However, the motion-corrected standing MRI used in this study provides less information than high-field systems and there is a substantial risk of missing small lesions or underestimating pathology [36]. Despite this, dispensing of the need for general anaesthesia facilitates follow-up examinations and alleviates the costs of the study. This technique provides reliable additional information on both bone components and soft tissues. Cartilage thickness was not assessed in this study due to partial volume artefacts and low image resolution, and a semi-quantitative system was chosen to evaluate the bones and soft tissue lesions.

The comparison of W-1 and W12 revealed a significant increase in synovial fluid total protein, total nucleated cell counts and CTX-II concentration in joints. In the light of published values encountered for severe inflammation [37] or variations observed in other models of animal OA [38,39], the degree of increase was low, as observed for other OA-associated changes in this study. High values of total protein concentration and total nucleated cell count encountered on W3 and W8 (Fig 8), might be linked to the surgical injury and progressive development of OA, as reported in the post-traumatic middle carpal joint OA model [38]. In the carpal joint model, another biomarker of cartilage degradation was used and revealed a similar pattern to that of CTX-II in this study. CTX-II has already been proven to be a sensitive and specific biomarker for early cartilage changes in an established canine model of experimental OA [39]. Finally, similarly to another published model of fetlock joint OA [15], no significant increase was observed in PGE2 concentration during the study period. This unexpected finding is not consistent with the results of the carpal osteochondral fragment model study and the fetlock osteochondral fragment-groove model [18], where a significant increase in PGE2 concentration in OA-affected joints was found compared to exercise-alone joints 10 or 11 weeks after surgery [14,38]. This could be attributed to the differences in time measurements or to the insufficient severity of lesions in this method. Nevertheless, postmortem evaluations revealed similar findings to those reported in the two previously cited models [18,38]. Indeed, macroscopic changes mostly included the presence of wear lines, while histological evaluation of osteochondral sections mainly revealed the presence of cartilage erosions, fibrocellular tissue and the formation of chondrocyte clusters. These findings support the relevance of the procedure presented in this study to induce OA-associated changes and underline the reliability of this protocol for use in the evaluation of new OA treatments in horses. Regarding post-mortem parameters, it should be acknowledged that the methods used for macroscopic and microscopic evaluation of the joints may have artificially lowered the total severity scores. Indeed, although the scoring system chosen for macroscopic lesions included 3 types of lesions, only 2 of these were present on the fetlocks observed in the study, as no palmar/plantar osteochondral disease was observed (Table 4). A single site was sampled for the microscopic evaluation of the joints, which might not be representative of all the structural changes throughout the joint. Post-mortem scores do not therefore provide an accurate representation of the true disease severity.

There are three main limitations of this study design that uses both front and both hind limbs. The first is the absence of a placebo control joint to compare the OA-induced lesions to a controlateral sham-operated joint. The second is that an objective evaluation of the degree of lameness of each limb was not possible, meaning that this parameter could not be considered in analysis, yet this pain-indicator symptom is important when evaluating OA and disease modifying therapies. The third main limitation of this study design is that all horses received intra-articular injections of stem cells on W3. The stem cells injected were from the same cell lines as those previously used in a safety study [19] and were not considered to have any relevant systemic effects in the analysis. The immunomodulatory properties of stem cells have been widely reported but their mechanisms of action have not been described in detail, research published to date has only focused on the general administration of stem cells. No studies have described potential systemic effects of stem cells after intra-articular administration, but such effects seem unlikely, given a reported reduction in trafficking of stem cells due to their size, which promotes passive cell entrapment [40]. The joint appears to be particularly prone to reducing cell trafficking due to the synovial membrane, which constitutes a real blood-joint barrier that limits the exchange of the synovial fluid and its content between the joint cavity and the bloodstream [41]. In addition, no adverse event occurred following stem cell injection that could have impaired the results of the study by shifting weight to another limb. A further limitation is that this study used a consensus agreement to limit the subjectivity of the semi-quantitative scores attributed. Although it is true that no repeatability tests were performed, the examiners rarely disagreed and in these cases, the images were reviewed and discussed carefully until consensus was reached. Finally, even if the number of horses used in the present study is comparable to similar studies performed in the field [12,15,18], the results of the statistical analyses should be considered with caution given the small sample size, but also given the lack of correction for multiple testing.

To conclude, surgical fragmentation of the proximal phalanx performed on both metacarpophalangeal and both metatarsophalangeal joints of experimental horses is a repeatable and well tolerated procedure that successfully induces mild OA-associated changes reflected by clinical, radiographic, ultrasonographic, MRI, biochemical, macroscopic and microscopic parameters. The few significant differences that were observed between fetlock positions (left, right, front or hind) would not preclude the use of such a model to evaluate the therapeutic effects of treatments. This model can be considered for use in efficacy studies in order to reduce the number of animals used and reduce individual variability of the evolution of OA-associated changes using each horse as its own control.

## Supporting information

S1 Dataset Lameness scores graded within 32 limbs on the day of stem-cell injection (before injection) on W3, the following day (D1), 3 days after injection (D3) and 7 days after injection (D7). (PDF)

S2 Dataset Scores and values of clinical, imaging, post-mortem and synovial fluid parameters within 32 fetlocks on weeks -1 and 3 and within 16 fetlocks on weeks 8 and 12. (PDF)

S1 Fig Arthroscopic images of the osteochondral fragmentation of the proximal phalanx. (a) Inspection of the dorsal aspect of the metacarpophalangeal joint. (b) Use of an 8 mm curved osteotome to create an osteochondral fragment of the dorso-proximo-medial border of the proximal phalanx. (c) Resulting fragment (arrow) attached to the joint capsule and defect bed (*). P1 –Proximal phalanx; Mc3 –Metacarpal condyle.
(PDF)

S2 Fig Open metacarpophalangeal joint of the right front fetlock of one horse showing locations of the surgically induced bone fragment and the osteochondral sample harvested for histological and immunohistological analysis performed on the medial part of the distal aspect of the metacarpal/tarsal condyle. (PDF)

S1 Table Magnetic resonance imaging parameters. (PDF)

S2 Table Results from the statistical analyzes (p values) comparing differences between W12 and W-1 values from the 16 fetlocks injected with placebo to evaluate variability in lesion evolution. (PDF)

S3 Table Mean (standard deviation) or median scores (1st quartile- 3rd quartile) for values measured in 32 fetlocks on weeks -1 and 3 and in 16 fetlocks on weeks 8 and 12. (PDF)
