# Supplementary material for: An experimentally induced osteoarthritis model in horses performed on both metacarpophalangeal and metatarsophalangeal joints: Technical, clinical, imaging, biochemical, macroscopic and microscopic characterization
Source: PLoS One. 2020 Jun 25;15(6):e0235251. doi: 10.1371/journal.pone.0235251 (PMC7316256; doi:10.1371/journal.pone.0235251)
Supplement: S1 Dataset — (PDF) [file pone.0235251.s001.pdf]

1: left fore
2: right fore
3: left hind
4: right hind

1: Placebo control
2: Stem cells

Horse number	Limb	Fetlock number	Treatment	Day post-injection	Lameness (grade/4)
1	1	11	2	0	0
1	1	11	2	1	0
1	1	11	2	3	0
1	1	11	2	7	0
1	2	12	1	0	0
1	2	12	1	1	0
1	2	12	1	3	0
1	2	12	1	7	0
1	3	13	1	0	0
1	3	13	1	1	0
1	3	13	1	3	0
1	3	13	1	7	0
1	4	14	2	0	0
1	4	14	2	1	0
1	4	14	2	3	0
1	4	14	2	7	0
2	1	21	2	0	0
2	1	21	2	1	0
2	1	21	2	3	0
2	1	21	2	7	0
2	2	22	1	0	0
2	2	22	1	1	0
2	2	22	1	3	0
2	2	22	1	7	0
2	3	23	2	0	0
2	3	23	2	1	0
2	3	23	2	3	0
2	3	23	2	7	0
2	4	24	1	0	0
2	4	24	1	1	0
2	4	24	1	3	0
2	4	24	1	7	0
3	1	31	1	0	1
3	1	31	1	1	0
3	1	31	1	3	0
3	1	31	1	7	0
3	2	32	2	0	0
3	2	32	2	1	0
3	2	32	2	3	0
3	2	32	2	7	0
3	3	33	2	0	0
3	3	33	2	1	0
3	3	33	2	3	0

3	3	33	2	7	0
3	4	34	1	0	1
3	4	34	1	1	1
3	4	34	1	3	1
3	4	34	1	7	2
4	1	41	1	0	0
4	1	41	1	1	0
4	1	41	1	3	0
4	1	41	1	7	0
4	2	42	2	0	0
4	2	42	2	1	0
4	2	42	2	3	0
4	2	42	2	7	1
4	3	43	2	0	0
4	3	43	2	1	0
4	3	43	2	3	0
4	3	43	2	7	0
4	4	44	1	0	0
4	4	44	1	1	0
4	4	44	1	3	0
4	4	44	1	7	0
5	1	51	1	0	0
5	1	51	1	1	0
5	1	51	1	3	0
5	1	51	1	7	0
5	2	52	2	0	0
5	2	52	2	1	1
5	2	52	2	3	1
5	2	52	2	7	1
5	3	53	1	0	0
5	3	53	1	1	0
5	3	53	1	3	0
5	3	53	1	7	0
5	4	54	2	0	0
5	4	54	2	1	2
5	4	54	2	3	1
5	4	54	2	7	1
6	1	61	2	0	1
6	1	61	2	1	1
6	1	61	2	3	0
6	1	61	2	7	0
6	2	62	1	0	0
6	2	62	1	1	0
6	2	62	1	3	0
6	2	62	1	7	0
6	3	63	1	0	2
6	3	63	1	1	2
6	3	63	1	3	1
6	3	63	1	7	1
6	4	64	2	0	0

6	4	64	2	1	0
6	4	64	2	3	0
6	4	64	2	7	0
7	1	71	1	0	1
7	1	71	1	1	0
7	1	71	1	3	0
7	1	71	1	7	0
7	2	72	2	0	0
7	2	72	2	1	0
7	2	72	2	3	0
7	2	72	2	7	1
7	3	73	2	0	0
7	3	73	2	1	0
7	3	73	2	3	0
7	3	73	2	7	0
7	4	74	1	0	0
7	4	74	1	1	0
7	4	74	1	3	1
7	4	74	1	7	1
8	1	81	1	0	0
8	1	81	1	1	0
8	1	81	1	3	0
8	1	81	1	7	0
8	2	82	2	0	0
8	2	82	2	1	0
8	2	82	2	3	0
8	2	82	2	7	0
8	3	83	2	0	0
8	3	83	2	1	0
8	3	83	2	3	0
8	3	83	2	7	0
8	4	84	1	0	0
8	4	84	1	1	0
8	4	84	1	3	0
8	4	84	1	7	0
