# Supplementary material for: An experimentally induced osteoarthritis model in horses performed on both metacarpophalangeal and metatarsophalangeal joints: Technical, clinical, imaging, biochemical, macroscopic and microscopic characterization
Source: PLoS One. 2020 Jun 25;15(6):e0235251. doi: 10.1371/journal.pone.0235251 (PMC7316256; doi:10.1371/journal.pone.0235251)
Supplement: S2 Dataset — (PDF) [file pone.0235251.s002.pdf]

Unstudied value
Not performed
Missing value

1: left fore
2: right fore
3: left hind
4: right hind

1: Placebo
2: Stem cells

-1: inclusion
0: surgery
3: treatment

Horse number	Limb	Fetlock number	Treatment	Week	Fetlock circumference (mean value)	Joint effusion (grade/4)	Lameness (grade/5)	US remodeling (grade/9)	RX remodeling (grade/9)	US synovitis (grade/4)	mri score (grade/27)	macroscopic score (grade/9)	Histologic score (grade/96)	Total protein (g/100 mL)	cells/µL	PGE2 (pg/mL)	CTX II (pg/mL)
1	1	11	2	-1	24,57	0	0	3	4	0	4			1,9	95	438,96	198,1
1	1	11	2	3	25,90	1	0	3	5	1	12			2,5	240	473,92	404,7
1	1	11	2	8													
1	1	11	2	12													
1	2	12	1	-1	24,37	0	0	1	1	0	4			1,8	75	578,25	173,1
1	2	12	1	3	25,60	1	0	2	1	1	8			2,3	260	318,39	363,9
1	2	12	1	8	24,60	0	0	4	1	1				1,8	130	393,11	222,3
1	2	12	1	12	24,63	0	0	5	1	1	9	1	18	2,2	147	251,05	198,6
1	3	13	1	-1	26,77	0	0	1	1	0	4			1,9	182	403,71	208,1
1	3	13	1	3	26,83	1	0	4	1	1	9			2,6	318	435,61	376,2
1	3	13	1	8	26,57	3	0	5	3	2				3,3	200		262,4
1	3	13	1	12	26,33	2	0	2	3	2	13	0	8	3,6	225		
1	4	14	2	-1	26,07	0	0	1	1	0	4			1,9	180	508,32	158
1	4	14	2	3	26,77	1	0	1	1	1	10			2,3	329	514,52	200,7
1	4	14	2	8													
1	4	14	2	12													
2	1	21	2	-1	26,20	0	0	2	0	0	2			1,6	124	414,51	231,8
2	1	21	2	3	27,00	1	0	2	0	0	4			2	607	578,25	299
2	1	21	2	8													
2	1	21	2	12													
2	2	22	1	-1	26,37	0	0	2	0	0	3			1,6	212	472,53	202,3
2	2	22	1	3	26,37	1	0	2	0	1	4			1,8	557	372,86	292,9
2	2	22	1	8	27,33	0	0	2	0	1				2,4	81	324,4	66,4
2	2	22	1	12	26,77	1	1	3	0	0	7	0	18	2,2	166	469,24	190
2	3	23	2	-1	27,33	0	0	1	1	0	7			1,8	319	416,7	94,7
2	3	23	2	3	28,70	2	0	3	1	3	8			2,3	357	446,48	253,3
2	3	23	2	8													
2	3	23	2	12													
2	4	24	1	-1	28,00	0	0	1	0	0	7			2	195	623,26	103,2
2	4	24	1	3	28,53	2	0	1	0	2	10			3,1	722	603,14	125,5
2	4	24	1	8	28,30	2	0	1	0	2				3,3	289	579,89	157,6
2	4	24	1	12	27,70	1	1	2	1	2	10	1	8	2,8	163		
3	1	31	1	-1	26,43	0	0	2	1	0	7			2	140	596,74	269,5
3	1	31	1	3	26,40	2	1	2	1	2	8			2,4	545	740,84	223,8
3	1	31	1	8	26,67	0	1	3	1	0				2,1	134	727,19	218
3	1	31	1	12	27,07	0	0	4	3	1	12	4	48	1,8	103	749,11	275,2
3	2	32	2	-1	26,40	0	0	2	0	0	7			2,1	141	446,48	194,6
3	2	32	2	3	26,50	2	0	3	0	2	10			2,8	307	636	212,4
3	2	32	2	8													

3	2	32	2	12													
3	3	33	2	-1	26,63	0	0	1	0	0	3			1,8	100	437,33	206,4
3	3	33	2	3	27,30	3	0	1	0	2	8			3,2	691	587,33	226,1
3	3	33	2	8													
3	3	33	2	12													
3	4	34	1	-1	26,67	0	0	0	0	1	3			1,8	99	268,77	155,2
3	4	34	1	3	27,83	3	1	0	0	2	10			3,4	746	394,5	213,8
3	4	34	1	8	27,90	4	1	2	1	2				4,2	489	489,86	485,6
3	4	34	1	12	27,70	4	0	3	1	1	12	1	12	2,6	248	453,35	587,3
4	1	41	1	-1	25,87	0	0	1	0	0	3			2	313	561,92	180,8
4	1	41	1	3	27,23	2	0	2	0	1	11			2,8	687	528,33	319
4	1	41	1	8	26,77	4	0	4	1	3				1,9	172	734,8	297,5
4	1	41	1	12	26,70	3	0	6	4	2	10	4	8	2	288	749,11	360,4
4	2	42	2	-1	26,00	0	0	1	0	0	3			2,3	281	646,64	205,3
4	2	42	2	3	27,07	2	0	2	0	2	9			3,4	1499	840,46	105,5
4	2	42	2	8													
4	2	42	2	12													
4	3	43	2	-1	26,67	0	0	1	0	0	6			2,4	75	1430,1	113,2
4	3	43	2	3	27,83	3	0	1	0	1	12			2,2	336	740,28	337,8
4	3	43	2	8													
4	3	43	2	12													
4	4	44	1	-1	26,90	0	0	1	0	0	5			2,4	68	564,71	140,6
4	4	44	1	3	28,50	2	0	1	0	1	9			2	166	266,81	372,2
4	4	44	1	8	27,63	1	0	2	0	1				3,4	38	219,64	64,9
4	4	44	1	12	27,37	2	0	2	0	1	6	0	24	2,2	193		153,3
5	1	51	1	-1	27,00	0	0	1	1	0	1			1,8	88	478,3	215,7
5	1	51	1	3	27,07	1	0	2	1	2	6			2,2	813	309,38	256,8
5	1	51	1	8	27,57	0	1	2	1	1				3,8	223	2345,41	353,8
5	1	51	1	12	27,37	0	0	4	2	0	7	0	32	2	145	829,57	331,5
5	2	52	2	-1	27,07	0	0	1	1	0	1			1,8	302		
5	2	52	2	3	27,13	1	0	2	1	0	7			2	734	315,39	189,7
5	2	52	2	8													
5	2	52	2	12													
5	3	53	1	-1	28,60	0	0	1	1	1	3			2	122	613,7	165,4
5	3	53	1	3	28,90	3	0	1	1	3	7			3	745	179,52	371,3
5	3	53	1	8	28,93	2	0	2	2	3				3	636	1536,25	73,5
5	3	53	1	12	29,33	3	1	4	3	1	5	4	8	2	403	5,94	207,6
5	4	54	2	-1	28,07	0	0	2	1	1	3			2	124	442,2	133,5
5	4	54	2	3	28,67	3	0	2	1	3	10			3,6	1066	388,22	242,7
5	4	54	2	8													
5	4	54	2	12													
6	1	61	2	-1	24,43	0	0	2	0	0	2			2,5	144	101,8	88,9
6	1	61	2	3	25,00	1	1	2	0	2	14			2,3	531	572,27	489
6	1	61	2	8													
6	1	61	2	12													
6	2	62	1	-1	24,63	0	0	2	0	0	1			2,5	55	81,69	51,2
6	2	62	1	3	25,37	0	0	2	0	2	6			2	359	464,61	355,2
6	2	62	1	8	25,93	1	0	2	2	1				3	107	624,29	155,2
6	2	62	1	12	25,77	1	0	4	3	1	6	3	16	2,5	441	426,79	204,4
6	3	63	1	-1	25,70	0	0	1	0	0	1			2	70	90,4	49,8

6	3	63	1	3	26,30	2	2	2	0	2	9			3	214	518,44	344,3
6	3	63	1	8	26,90	2	1	5	7	3				3,6	167	613,65	288,5
6	3	63	1	12	26,50	2	1	6	7	2	11	3	24	2,3	140	431,17	929,5
6	4	64	2	-1	25,80	0	0	1	1	0	2			2	70	112,8	64,6
6	4	64	2	3	26,17	0	0	2	1	2	7			2	341	434	247,9
6	4	64	2	8													
6	4	64	2	12													
7	1	71	1	-1	26,03	0	0	1	0	0	1			2	206	373,47	189,7
7	1	71	1	3	26,83	2	1	3	0	2	5			2,6	162	866,81	290,4
7	1	71	1	8	27,47	2	1	7	4	3				2,9	330	494,52	370,8
7	1	71	1	12	27,10	2	1	7	7	3	8	5	12	2,4	168	386,04	453
7	2	72	2	-1	26,73	0	0	2	0	0	2			2	91	398,75	156,2
7	2	72	2	3	26,83	2	0	3	0	0	9			3,2	2524	527,76	169,4
7	2	72	2	8													
7	2	72	2	12													
7	3	73	2	-1	28,33	0	0	0	0	0	1			2	65	386,11	172,95
7	3	73	2	3	28,80	2	0	1	0	1	6			3	351	396,62	260,2
7	3	73	2	8													
7	3	73	2	12													
7	4	74	1	-1	27,83	0	0	2	0	0	1			2	68	228,9	134,2
7	4	74	1	3	28,17	1	0	3	0	2	5			3,3	415	597,1	197,8
7	4	74	1	8	28,63	4	2	6	3	4				4,6	1585	774,71	178,4
7	4	74	1	12	29,03	4	0	6	6	3	9	3	16	2,5	267	369,3	422,8
8	1	81	1	-1	24,90	0	0	1	0	0	2			1,8	25	130,35	49,3
8	1	81	1	3	25,63	1	0	3	0	1	8			2,2	107	251,11	272,9
8	1	81	1	8	26,40	1	0	3	1	2				3,4	138	418,09	1593,2
8	1	81	1	12	26,10	1	0	4	2	1	9	1	24	2,2	77	209,74	84,8
8	2	82	2	-1	25,20	0	0	2	0	0	2			2	93	112,15	55
8	2	82	2	3	25,73	1	0	2	0	1	8			2,6	224	221,62	191,6
8	2	82	2	8													
8	2	82	2	12													
8	3	83	2	-1	26,47	0	0	3	0	0	1			2	58	346,65	40,4
8	3	83	2	3	26,73	2	0	3	0	2	5			3	178	266,81	109,4
8	3	83	2	8													
8	3	83	2	12													
8	4	84	1	-1	26,13	0	0	3	0	0	0			2	16	367,23	62,6
8	4	84	1	3	26,80	2	0	3	0	2	6			3,3	152	251,11	132,5
8	4	84	1	8	27,47	2	0	3	0	2				2,8	77	125,85	268,7
8	4	84	1	12	27,17	2	0	4	0	2	8	3	16	2,3	210	308,32	181,2
