# Supplementary material for: An experimentally induced osteoarthritis model in horses performed on both metacarpophalangeal and metatarsophalangeal joints: Technical, clinical, imaging, biochemical, macroscopic and microscopic characterization
Source: PLoS One. 2020 Jun 25;15(6):e0235251. doi: 10.1371/journal.pone.0235251 (PMC7316256; doi:10.1371/journal.pone.0235251)
Supplement: S1 Fig — (a) Inspection of the dorsal aspect of the metacarpophalangeal joint. (b) Use of an 8 mm curved osteotome to create an osteochondral fragment of the dorso-proximo-medial border of the proximal phalanx. (c) Resulting fragment (arrow) attached to the joint capsule and defect bed (*). P1 –Proximal phalanx; Mc3 –Metacarpal condyle. (PDF) [file pone.0235251.s003.pdf]

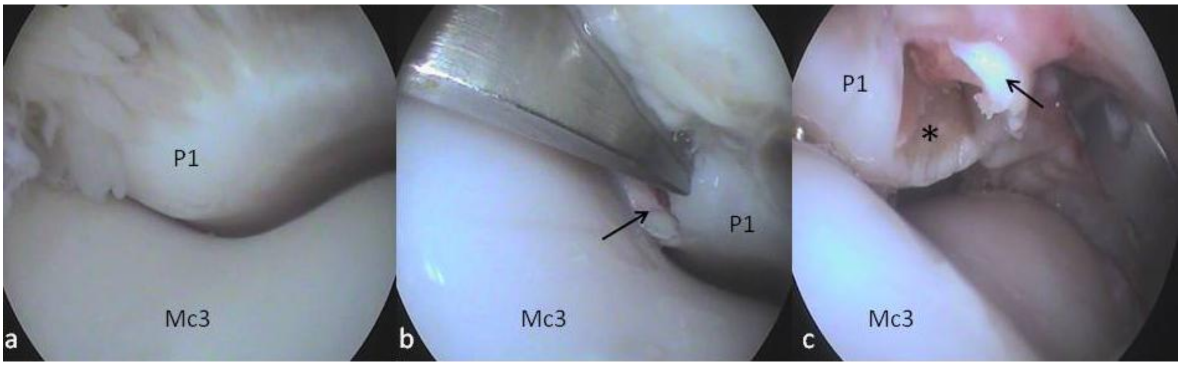

S1 Fig. Arthroscopic images of the osteochondral fragmentation of the proximal phalanx. (a) Inspection of the dorsal aspect of the metacarpoc-phalangeal joint. (b) Use of a 8 mm curved osteotome to create an osteochondral fragment of the dorso-proximo-medial border of the proximal phalanx. (c) Resulting fragment (arrow) attached to the joint capsule and defect bed (*). P1 – Proximal phalanx; Mc3 – Metacarpal condyle
