# Supplementary material for: An experimentally induced osteoarthritis model in horses performed on both metacarpophalangeal and metatarsophalangeal joints: Technical, clinical, imaging, biochemical, macroscopic and microscopic characterization
Source: PLoS One. 2020 Jun 25;15(6):e0235251. doi: 10.1371/journal.pone.0235251 (PMC7316256; doi:10.1371/journal.pone.0235251)
Supplement: S2 Fig — (PDF) [file pone.0235251.s004.pdf]

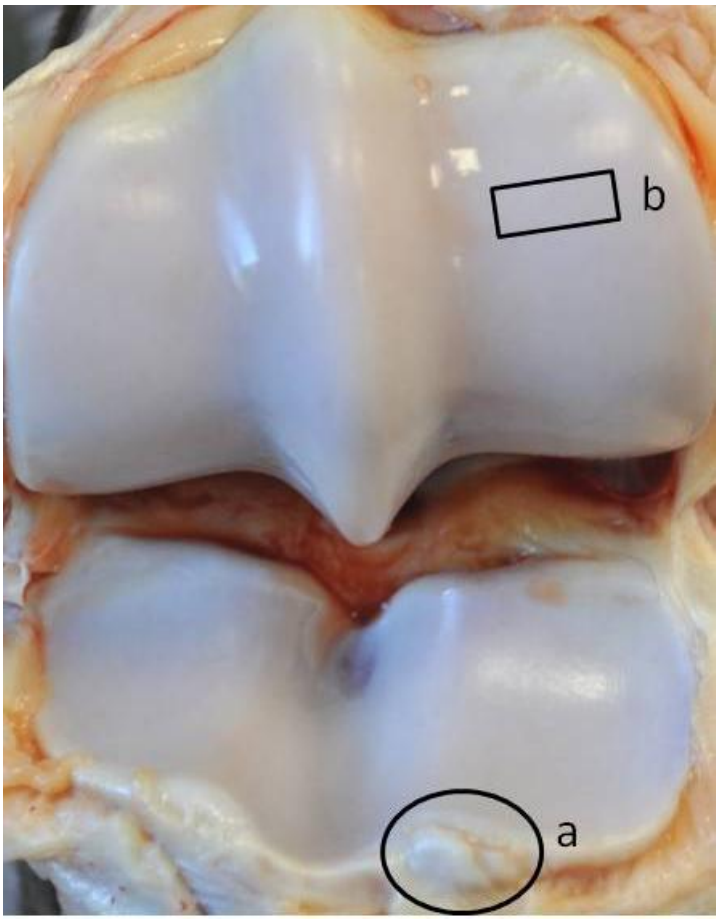

S2 Fig. Open metacarpophalangeal joint of the right front fetlock of one horse showing locations of the surgically induced bone fragment (a) and the osteochondral sample (b) harvested for histologic and immunohistologic analysis performed on the medial part of the distal aspect of the metacarpal/tarsal condyle.
