# Supplementary material for: An experimentally induced osteoarthritis model in horses performed on both metacarpophalangeal and metatarsophalangeal joints: Technical, clinical, imaging, biochemical, macroscopic and microscopic characterization
Source: PLoS One. 2020 Jun 25;15(6):e0235251. doi: 10.1371/journal.pone.0235251 (PMC7316256; doi:10.1371/journal.pone.0235251)
Supplement: S1 Table — (PDF) [file pone.0235251.s005.pdf]

S1 Table. Magnetic Resonance Imaging Parameters

**Sequence**	**Acquisition type**	**Slice thickness/ interslice gap** (mm)	**TE** (msec)	**TR** (msec)	**FOV**	**FA**	**Pixel Band Width**
**T1-GRE UFAST**	2D	5.0/1.0	8	52	160x160	50	49
**T1-GRE HRUFAST**	2D	3.0/1.0	8	52	180x180	50	49
**T2-FSE MI**	2D	5.0/1.0	88	1544	175x175	90	65
**STIR-FSE MI**	2D	5.0/1.0	22	2536	175x 175	90	65

TE – echo time; TR – repetition time; FA – flip angle; FOV – field of view; GRE – gradient echo; FSE – fast spin echo; STIR – short tau inversion recovery; MI – motion insensitive
