# Supplementary material for: An experimentally induced osteoarthritis model in horses performed on both metacarpophalangeal and metatarsophalangeal joints: Technical, clinical, imaging, biochemical, macroscopic and microscopic characterization
Source: PLoS One. 2020 Jun 25;15(6):e0235251. doi: 10.1371/journal.pone.0235251 (PMC7316256; doi:10.1371/journal.pone.0235251)
Supplement: S2 Table — (PDF) [file pone.0235251.s006.pdf]

S2 Table. Results from the statistical analyzes (p values) comparing differences between W12 and W-1 values from the 16 fetlocks injected with placebo to evaluate variability in lesion evolution

Values resulting from the differences between W12 and W-1 values (or W12 values alone of post-mortem scores) of the 16 fetlocks injected with placebo were compared:

- between the 8 horses to evaluate interindividual variability in the development of OA-associated lesions,
- between pooled data of all 8 individuals for each fetlock position (fore, hind, right and left) to evaluate operated limb variability.

* Indicates significant effects with $p < 0.05$.

	Joint circumference (cm)	Joint effusion (grade/4)	Ultrasound osteophytes (grade/9)	Radiographic osteophytes (grade/9)	Ultrasound synovitis (grade/4)	MRI score (grade/27)	Macroscopic score (grade/18)	Microscopic score (grade/96)	Total protein (g/100 mL)	Total nucleated cells/µL	PGE2 (pg/mL)	CTX II (pg/mL)
Between 8 horses	0.08	0.75	0.35	0.24	0.19	0.47	0.57	0.94	0.28	0.55	0.65	0.17
Between 8 Left *and* 8 Right pooled fetlocks	1,00	0.91	0.12	**0.04***	0.83	0.40	0.21	0.87	0.84	0.21	0.86	0.59
Between 8 Fore *and* 8 Hind pooled fetlocks	0.75	**0.02***	0.30	0.83	0.44	0.83	0.67	0.18	0.22	0.24	0.60	0.20
