# Supplementary material for: An experimentally induced osteoarthritis model in horses performed on both metacarpophalangeal and metatarsophalangeal joints: Technical, clinical, imaging, biochemical, macroscopic and microscopic characterization
Source: PLoS One. 2020 Jun 25;15(6):e0235251. doi: 10.1371/journal.pone.0235251 (PMC7316256; doi:10.1371/journal.pone.0235251)
Supplement: S3 Table — (PDF) [file pone.0235251.s007.pdf]

S3 Table. Mean (standard deviation) or median scores (1st quartile- 3rd quartile) for values measured in 32 fetlocks on weeks -1 and 3 and in 16 fetlocks on weeks 8 and 12.

	Week	Joint circumference (cm)	Joint effusion (grade/4)	Ultrasound osteophytes (grade/9)	Radiographic osteophytes (grade/9)	Ultrasound synovitis (grade/4)	MRI score (grade/27)	Macroscopic score (grade/18)	Microscopic score (grade/96)	Total protein (g/100 mL)	Total nucleated cells/µL	PGE2 (pg/mL)	CTX II (pg/mL)
**Total**	**-1**	26.4 (1.1)	0 (0-0)	1 (1-2)	0 (0-1)	0 (0-0)	3 (1-4)			2 (1.8-2)	99 (70-181)	417 (308-535)	156 (92-196)
	**3**	27.0 (1.1)	2 (1-2)	2 (1.5-3)	0 (0-1)	2 (1-2)	8 (6.5-10)			2.6 (2.2-3.1)	359 (284-707)	441 (316-561)	253 (199-341)
	**8**	27.2 (1.1)	2 (0.8-2.3)	3 (2-4.2)	1 (0.7-2.2)	2 (1-3)				3.1 (2.7-3.5)	169 (124-299)	580 (406-731)	242 (157-312)
	**12**	27.0 (1.1)	2 (1-2.3)	4 (3-5.2)	2.5 (1-3.2)	1 (1-2)	9 (7-10.2)	2 (0.7-3.2)	16 (11-24)	2.2 (2.2-2.5)	180 (147-253)	427 (308-469)	241 (192-407)
**Fore fetlocks**	**-1**	25.8 (0.9)	0 (0-0)	2 (1-2)	0 (0-1)	0 (0-0)	2 (1.7-3.2)			2 (1.8-2)	132 (90-208)	439 (252-520)	190 (123-204)
	**3**	26.3 (0.7)	1 (1-2)	2 (2-2.2)	0 (0-1)	1 (0.7-2)	8 (6-9.2)			2.3 (2.1-2.7)	545 (284-711)	474 (317-575)	273 (202-337)
	**8**	26.6 (1.0)	0.5 (0-1.3)	3 (2-4)	1 (1-1.2)	1 (1-2.2)				2.6 (2.1-3.1)	136 (124-185)	559 (412-729)	260 (202-358)
	**12**	26.4 (0.9)	1 (0-1.3)	4 (4-5.2)	2.5 (1.7-3.2)	1 (0.7-1.2)	8.5 (7-9.2)	2 (0.7-4)	18 (15-26)	2.2 (2-2.3)	156 (135-198)	448 (352-749)	240 (196-339)
**Hind fetlocks**	**-1**	27.0 (0.9)	0 (0-0)	1 (1-1.2)	0 (0-1)	0 (0-0)	3 (1-4.2)			2 (1.9-2)	87 (68-138)	410 (327-522)	134 (87-160)
	**3**	27.7 (1.0)	2 (1.8-3)	1.5 (1-3)	0 (0-1)	2 (1-2)	8.5 (6.7-10)			3 (2.3-3.2)	346 (292-699)	434 (328-516)	245 (200-339)
	**8**	27.8 (0.8)	2 (2-3.3)	2.5 (2-5)	1.5 (0-3)	2 (2-3)				3.3 (3.2-3.8)	244 (245-526)	580 (355-694)	220 (137-274)
	**12**	27.6 (1.1)	2 (2-3.3)	3.5 (2-4.5)	2 (0.7-3.7)	2 (1-2)	9.5 (7.5-11.2)	2 (0.7-3)	14 (8-18)	2.4 (2.3-2.7)	217 (186-253)	369 (308-431)	315 (188-546)
**Left fetlocks**	**-1**	26.3 (1.2)	0 (0-0)	1 (1-2)	0 (0-1)	0 (0-0)	2.5 (1-4)			2 (1.8-2)	111 (74-154)	416 (367-499)	177 (93-207)
	**3**	27.0 (1.1)	2 (1-1)	2 (1.5-3)	0 (0-1)	2 (1-2)	8 (6.5-10)			2.5 (2.3-3)	357 (279-647)	474 (353-575)	299 (255-358)
	**8**	27.1 (0.8)	2 (0.8-2.3)	3.5 (2.7-5)	1.5 (1-3.2)	2.5 (1.7-3)				3.1 (2.7-3.5)	186 (160-250)	727 (554-1136)	293 (251-358)
	**12**	27.1 (1.0)	2 (0.8-2.3)	4 (4-6)	3 (2.7-4.7)	1.5 (1-2)	9.5 (7.7-11.2)	3.5 (0.7-4)	18 (8-26)	2.1 (2-2.1)	156 (131-157)	431 (298-431)	331 (241-332)
**Right Fetlocks**	**-1**	26.4 (1.1)	0 (0-0)	1.5 (1-2)	0 (0-1)	0 (0-0)	3 (1.7-4)			2 (1.9-2)	96 (70-184)	442 (249-537)	141 (84-166)
	**3**	27.0 (1.1)	1.5 (1-2)	2 (1.7-2.2)	0 (0-1)	2 (1-2)	8.5 (6.7-10)			2.7 (2-3.3)	387 (295-737)	394 (317-521)	207 (185-259)
	**8**	27.2 (1.3)	1.5 (0.8-2.5)	2 (2-3.2)	0.5 (0-1.2)	1.5 (1-2)				3.1 (2.7-3.6)	118 (80-339)	441 (298-591)	168 (133-234)
	**12**	27.1 (1.3)	1.5 (1-2.5)	3.5 (2.7-4.2)	1 (0-1.5)	1 (1-2)	8.5 (6.7-9.2)	1 (0.7-3)	16 (15-18)	2.4 (2.2-2.5)	201 (165-253)	398 (324-447)	199 (186-314)
